# Learning transport processes with machine intelligence

**DOI:** 10.1038/s41598-022-15416-y

**Published:** 2022-07-09

**Authors:** Francesco Miniati, Gianluca Gregori

**Affiliations:** Department of Physics, University of Oxford, Parks Road, Oxford, OX1 3PU UK

**Keywords:** Astronomy and astrophysics, Fluid dynamics, Plasma physics, Computational science

## Abstract

Transport processes ruled by complex micro-physics and impractical to theoretical investigation may exhibit emergent behavior describable by mathematical expressions. Such information, while implicitly contained in the results of microscopic-scale numerical simulations close to first principles or experiments is not in a form suitable for macroscopic modelling. Here we present a machine learning approach that leverages such information to deploy micro-physics informed transport flux representations applicable to a continuum mechanics description. One issue with deep neural networks, arguably providing the most generic of such representations, is their noisiness which is shown to break the performance of numerical schemes. The matter is addressed and a methodology suitable for schemes characterised by second order convergence rate is presented. The capability of the methodology is demonstrated through an idealized study of the long standing problem of heat flux suppression relevant to fusion and cosmic plasmas. Symbolic representations, although potentially less generic, are straightforward to use in numerical schemes and theoretical analysis, and can be even more accurate as shown by the application to the same problem of an advanced symbolic regression tool. These results are a promising initial step to filling the gap between micro and macro in this important area of modeling.

## Introduction

Conservation laws are fundamental laws of physics reflecting underlying symmetries of nature^1^. In continuum mechanics they apply in a Lorentz invariant local form and are formulated mathematically as the continuity equation

1 $$\begin{aligned} \frac{\partial u}{\partial t} = -\nabla \cdot {\mathbf{q}}, \end{aligned}$$

where *u*, is the volume density of the conserved variable and ${\mathbf{q}}(u)$ the corresponding current or flux density. Equation (1) states that the rate of change of a conserved variable within a volume *V* is due to its flux across the volume’s surface, $\partial V$:

2 $$\begin{aligned} \frac{d}{dt} \int _{V} u\, dV = \int _{\partial V} {\mathbf{q}} \cdot d{\mathbf{s}}. \end{aligned}$$

The continuity equation, typically in the form of a system of coupled partial differential equations (PDEs) for a state vector of conserved variables, *u*, appears virtually in all fields of modern science and engineering where it is employed particularly for the description of fundamental phenomena related to fluids, plasmas, and solids. The ability to obtain high fidelity models based on its accurate solution is therefore of great interest. Given its nonlinear character, the use of advanced numerical integration techniques for hyperbolic systems is usually required, which has given rise to a now well established and mature field of applied mathematics^2–4^.

However, the accurate knowledge of the transport term, ${\mathbf{q}}$, entering the continuity equation is generally missing, particularly for diffusive processes which depend on complex physics mechanisms operating at microscopic scales. This applies to many fundamental and industrial applications, including fusion^5–9^ and cosmic plasmas^10–12^, liquid metals^13,14^, hypersonic flows during spacecraft re-entry^15^, semiconductor devices^16,17^, phononic transport in solids^18,19^. One outstanding example is the case of heat transport. For classical ideal fluids and gases it is well established that the heat flux is proportional to the temperature gradient as collisions between nearby particles enforce a local energy flow from hotter to colder regions. Thus Spitzer–Härm theory^20^ gives Fick’s law, ${\mathbf{q}}=-\kappa \nabla T$, where $\kappa$ is the coefficient of thermal conduction and *T* the temperature. However, it has long been realised that the ideal gas approximation breaks down when the electron mean free path approaches or exceeds the temperature gradient scale-length, $L_{T}$, a condition common in thermonuclear fusion plasmas^5,7^. Modified versions of Fick’s law have been proposed in the literature but are often very poor and fail to generalize^6,7^.

While such processes can in principle be modeled by integrating sets of microscopic (e.g. kinetic) equations progressively closer to first principles (which are, however, impractical to model macroscopic systems), this capability has unfortunately not yet translated into the formulation of transport terms, ${\mathbf{q}}$, employable in a continuum mechanics description, suitable for modeling macroscopic systems. In addition, there are physical conditions under which even current kinetic or ab initio codes do not provide consistent results^21^. In this case it would be desirable to have the ability to learn about such transport terms directly from actual experimental data.

In this paper we describe a machine learning (ML) based approach designed to improve our modeling capability and theoretical understanding of generic transport processes by learning directly from data provided either by microscopic-scale numerical simulations or even experiments. In particular, we apply deep learning techniques to obtain a representation of the transport process as a function of the state vector.

In the past decade artificial intelligence has emerged as a powerful technology^22^ and there has been great interests in its use for scientific applications in general^23–28^. In the context of computational fluid dynamics machine learning has been leveraged as an accelerator, i.e., in order to enhance the performance of numerical solvers. In particular, we have seen the development of powerful emulators, i.e. machines capable of fully representing PDE solvers in order to reproduce the results of conventional numerical simulation codes but at a significantly lower computational cost and/or higher accuracy^29–35^. Alternatively, researchers have focused on augmenting the modeling capability of numerical methods. Here one typically employs a learnable function to assist or replace altogether modular components of the numerical scheme, particularly those most affected by finite resolution effects, so as to enhance the overall performance of the method^36–41^. In the context of phononic thermal transport ML techniques have been applied to generate accurate effective (force field) potentials from high-fidelity density functional theory simulations^19,42^. In the spirit of the augmented methods discussed above, these effective potentials are then used in ab initio molecular dynamics simulations to predict heat conduction in new materials^19,43,44^. Note that pure ML emulators are not formulated on the basis of numerical analysis. While as a result these solvers may be more flexible and powerful as they are not subject to mathematical constrains as numerical methods for hyperbolic systems (e.g., the Courant–Friedrichs–Lewy condition), they usually come short of the stability, generalisation and robustness characterising full fledged numerical methods^2–4^. These properties tend to be better preserved in the augmented methods.

Our aim here is aligned with the development of augmented methods in that we employ deep learning techniques to ultimately improve the accuracy of numerical simulation models. At the same time our scope is different in that our target is not a representation of the optimal numerical scheme, rather a representation of the unknown underlying transport physics, somewaht similar to the above phononic application. Proper modeling of the latter usually requires a microscopic description based on a different (and much more expensive) computational approach or, at times, even experiments. Our method therefore can also be seen as an accelerator in that it deploys a micro-physics informed transport term usable in a conventional fluid approach without incurring the cost of a full microscopic description. As will be shown in the next section, however, a latent representation of a transport process provided by a deep neural network has limited smoothness properties, which breaks the performance of a numerical scheme. For second order accurate schemes the issue can be addressed by using latent representations of the flux gradient instead of the flux function itself. This approach can be extended for higher order schemes but at the cost of higher complexity. In any case, data regularization becomes now necessary to ensure a reliable estimate of the gradients from the generally noisy data.

On an even more ambitious scale, one can attempt to obtain symbolic representation of the transport process through a symbolic regression analysis of the data. A mathematical expression is of great theoretical importance and allows for the potential discovery of new physical relational laws^45,46^. In addition, it is straightforward to use in a numerical scheme. So would it be the ultimate solution to the problem. Unfortunately, however, in general this task is significantly more difficult and potentially strongly affected by the quality of the data^46^.

In the remainder of this paper we first present in detail our proposed method to learn representations of transport processes, addressing the numerical issues, the the data regularization procedure and the employed deep learning methods. In the second part we demonstrate the viability of the method in a scenario of real scientific interest, the case of heat transport in a high temperature plasma previously discussed, including the application of an advanced symbolic regression tool.

## Methodology

### Preliminaries

Building a ML model of a transport process requires a set of data representing the solution to the associated Eq. (1). The data can be obtained either through direct measurements (experiments), numerical simulations with sufficient modeling capability, or both. In the former case the data most likely represents time variations of the conserved quantity and in the latter case values of the flux density itself. Obviously the data should only contain information about the transport process of interest. For example, in studying diffusion the effects of advection must be subtracted out. More precisely, our data is laid out on a discrete grid, $\Gamma \in {\mathbb{Z}}$, defined in a one-dimensional spacial domain $[0,L]\in {\mathbb{R}}$. This simple setting is not restrictive in terms of information scope and conveniently keeps the data complexity to a minimum. Given the mesh spacing, *h*, we define a set of control volumes $i \in \Gamma$ corresponding to region of space $[i\,h,(i+1)\,h]$ with boundaries belonging to a face-centered discretisation space based on those control volumes: $\{\Gamma ^{e} = i + 1/2 : i \in \Gamma \}$. It is convenient to relate the time variation of the conserved quantity and the fluxes by integrating Eq. (1) in a space-time slab ($h, \delta t$)^47^

3 $$\begin{aligned} {\bar{q}}_{i+\frac{1}{2}}-{\bar{q}}_{i-\frac{1}{2}} = - \frac{h}{\delta t} \delta {\bar{u}}_{i}, \end{aligned}$$

where ${\bar{q}}$ is the time averaged flux over $\delta t$, and $\delta {\bar{u}}_{i}$ is the space averaged time variation of *u* inside the *i*-th control volume during $\delta t$. Note that Eq. (3) at this stage is exact. Then knowing the value of the flux *q* at one of the boundaries, $b=0,L$, one can write:

4 $$\begin{aligned} {\bar{q}}_{k\pm\frac{1}{2}} = q_{b} \mp \frac{h}{\delta t}\sum _{i=m}^{n} \delta {\bar{u}}_{i} \end{aligned}$$

where we use the upper sign and set $(m, n, b)=(0,k,0)$ if the flux value is known at the domain’s origin and use the lower sign and set  *(m,m,b)=(k*, *L*, *L*) if the flux value is known at the domain’s end. Equation (4) is useful when the value of the flux, ${\bar{q}}_{i+1/2}$’s, is not directly measurable, in which case it can still be inferred from the $\delta {\bar{u}}_{i}$’s. It also makes it clear that the fluxes and time variations we deal with are actually time and volume averages not instantaneous or point-wise values. Similarly, although we use a one-dimensional model, experimental data will require a surface averaging of the flux. Since in general such experimental and numerical inaccuracies can be quantified there is in principle control over the quality of the data and the inferred model. With this understanding the data, either the set of $\delta {\bar{u}}_{i}$’s or equivalently of ${\bar{q}}_{i+1/2}$’s, can be used to construct the labels for supervised training. We address details related to this step next.

### Smoothness

The performance of a numerical scheme depends on the smoothness of the functions appearing in the target PDEs. While this is not an issue with analytic expressions unless the functions of interest are intrinsically irregular, the question arises in the case of latent representations provided by a Multilayer-Perceptron (MLP), owing to their noisy character. The smoothness we are referring to here concerns the validity of Taylor expansion’s approximation, from which the accuracy and convergence properties of a scheme are inferred through the methods of numerical analysis^3^. We thus assess the smoothness of an MLP function by the residual error, ${\mathcal{E}}_{p}$, of its Taylor expansion up to order $p-1$, for variation *h* of an individual components $\xi$ of ${\mathbf{x}}$, namely

5 $$\begin{aligned} {\mathcal{E}}_{p} (h; {\mathbf{x}}_{0}, f)=\frac{1}{f_{0}} \left( f(\mathbf{x}_{0}+h{\hat{{\varvec{\xi }}}})-\sum _{k=0}^{p-1} \frac{\partial ^{k}_{\xi } f_{0}}{k!} h^{k} \right) = O(h^{p}) \end{aligned}$$

where ${\mathbf{x}}_{0}$ is the expansion point, $\partial ^{k}_{\xi } f_{0}\equiv \partial ^{k}_{\xi } f({\mathbf{x}}_{0})$ and ${\hat{{\varvec{\xi }}}}$ is a unit vector in the $\xi$-direction of parameter space. The last equality is expected to hold for any *p* for infinitely differentiable functions such as the ones we are dealing with. We compute ${\mathcal{E}}_{p}$ for the analytic function $q({\mathbf{x}}=(n,~T,~\beta ))$ defined in Eq. (13), and for its MLP representation described in the ‘MLP Representation of the Flux Function’ in Methods, although the specific details do not affect the conclusions of the current discussion.

**Figure 1:**
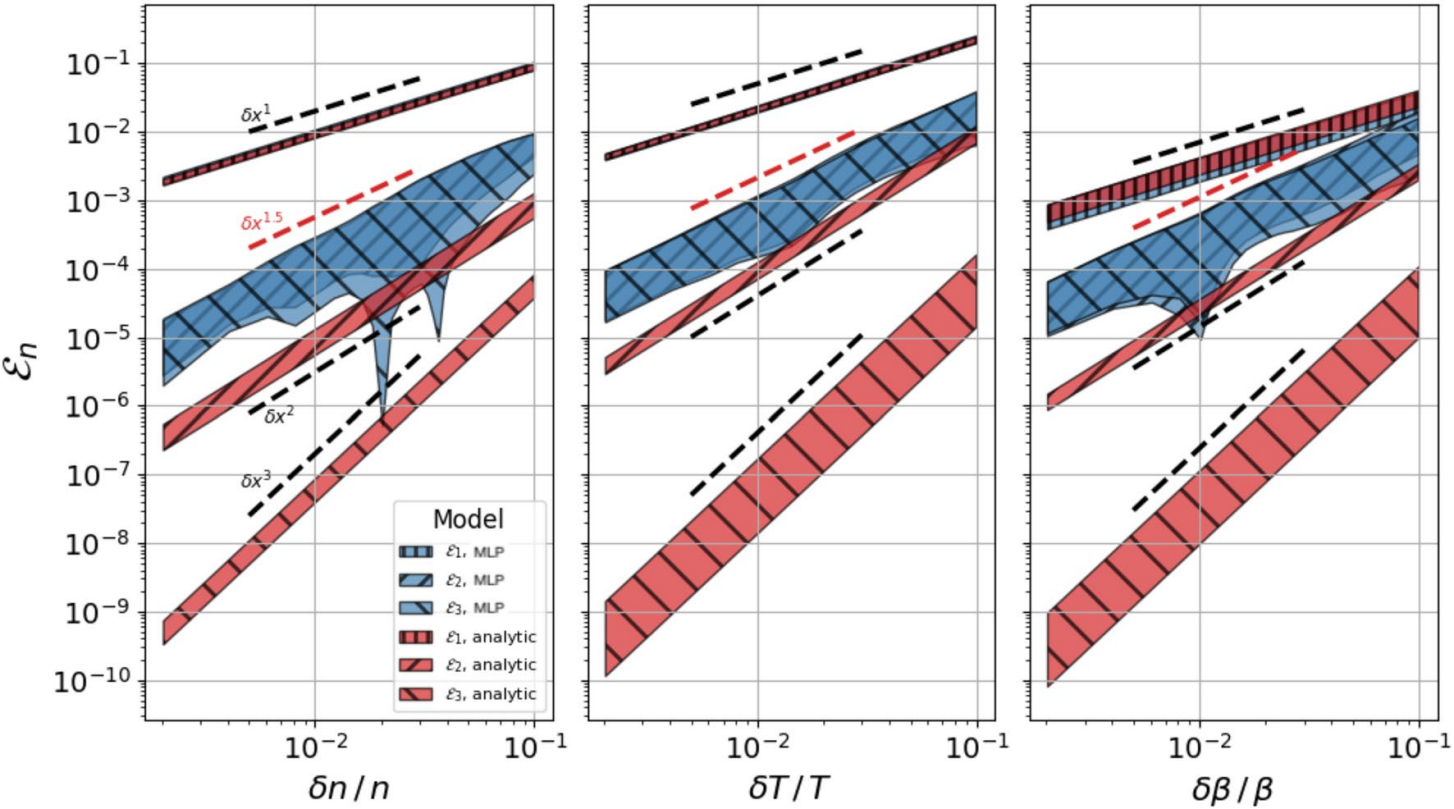
Taylor expansion’s error: residual error from Taylor expansion up to order 0, 1, 2 (corresponding to ${\mathcal{E}}_{p}$ with $p=1, 2, 3$, respectively in the legend) of the analytic flux function *q* in Eq. (13) (red) and its MLP representation (blue). Each panel corresponds to variations of each individual thermodynamic variable, with the shaded regions representing the range between the absolute of the mean value (bottom boundary) and one standard deviation (upper boundary) for a sample of 30 randomly chosen expansion points.

In Fig. 1 we plot ${\mathcal{E}}_{p}(h)$ as a function of $\delta \xi /\xi \equiv h/x_{0,\xi }$, after averaging over 30 expansion points, ${\mathbf{x}}_{0}$, randomly chosen in the domain of the *q* function for $p=1,~2,~3$. Each panel corresponds to the expansion along a different component $\xi$, with the shaded regions representing the range between the absolute mean value (bottom boundary) and one standard deviation (upper boundary). The results indicate that while for the analytic case (red), ${\mathcal{E}}_{p}\propto h^{p}$, as expected, for the MLP representation (blue) only ${\mathcal{E}}_{1}$ follows expectations, in fact overlapping with the analytic counterpart. Instead, ${\mathcal{E}}_{2}$ scales only as $h^{1.5}$ and ${\mathcal{E}}_{3}$ is virtually equivalent to ${\mathcal{E}}_{2}$ (notice the cross hatch pattern), suggesting that additional terms of Taylor’s series do not improve the approximation.

**Figure 2:**
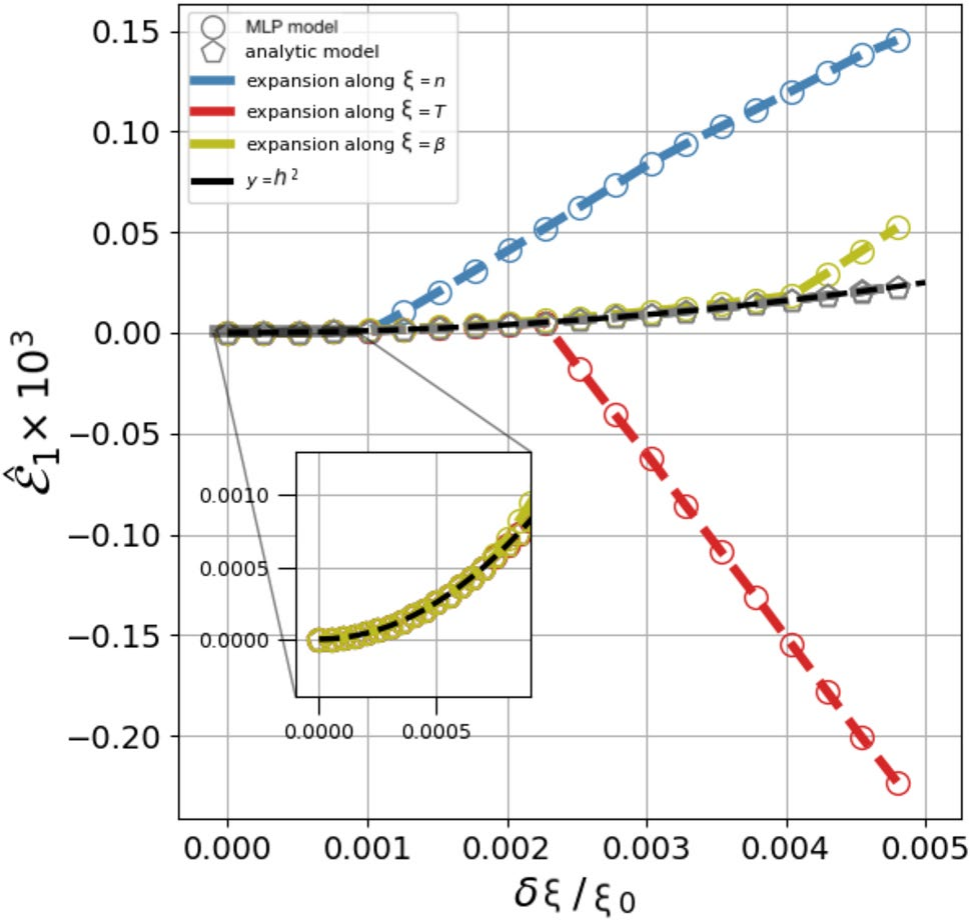
Smoothness test: plot of the scaled residual of a first order Taylor expansion ${\hat{{\mathcal{E}}}}_{1}= {\mathcal{E}}_{2} \,/\,{\mathcal{E}}_{2}(h\approx 10^{-3}x_{0,\xi })$, with respect to each thermodynamic variable, $n, T, \beta$ (no sample averaging over the expansion point was computed). The open gray pentagons represent the three overlapping expansions for the analytic flux function *q* in Eq. (13). The blue, red and olive symbols (circles + dash-line) show the expansions with respect to $n, T, \beta$, respectively, for the case of the MLP representation. The black solid line is the parabolic curve that all scaled residual errors are expected to follow, an expectation fulfilled by the analytic case, but not the MLP representation, except that for a very short interval.

In Fig. 2 we plot ${\hat{{\mathcal{E}}}}_{2}$, i.e. ${\mathcal{E}}_{2}$ computed for a single value ${\mathbf{x}}_{0}$ but rescaled by the factor ${\mathcal{E}}_{2}(h\approx 10^{-3}\,x_{0,\xi })$. The figure shows that the scaled Taylor expansion of the MLP representation does follow the parabolic curve (black line), but only within a much smaller interval than the analytic case (gray pentagon). In the specific case, beyond $\delta \xi /\xi \le 10^{-3}$, ${\hat{{\mathcal{E}}}}_{2}$ grows linearly indicating that the function’s derivative has changed substantially. That this simple description, indicative of a noisy character, is sufficient to reproduce the qualitative and quantitative behaviour of the MLP representation illustrated in Supplementary Fig. S1. Here the same sample averages of ${\mathcal{E}}_{p}$ as in Fig. 1 are plotted together with those of the analytic function *q* modified for an additional random term proportional to its first derivative, namely

6 $$\begin{aligned} q_{s}({\mathbf{x}}) = q({\mathbf{x}}) + a\, \nabla q \, \frac{h^{1.5}}{x_{0,\xi }^{0.5}}, \end{aligned}$$

where *a* is a random number sampled uniformly within $[-A, ~A]$ with *A* of order unity.

This lack of smoothness implies poor numerical performance. For example, applying a finite difference centered scheme to estimate the gradient of *q* yields only $O(h^{0.5})$ accuracy as opposed to $O(h^{2})$ as usual. The resulting truncation error is likewise of order $\tau (h)\propto O(h^{0.5})$. Oddly enough, however, due to the stochastic character of the offending term spoiling Taylor expansion’s approximation the convergence rate would be better than inferred by the truncation error because the error accumulates only as $N_{steps}^{1/2}$. Therefore, in a finite different scheme for example, at a given solution time, $t=N_{\mathrm{steps}}\;\Delta t$ and for fixed $\Delta x /\Delta t =$const.

7 $$\begin{aligned} \varepsilon (t,\Delta x, \Delta t)= \sum _{N_{\mathrm{steps}}} \tau (\Delta x)\; \Delta t \propto \tau (\Delta x)\; (\Delta t)^{1/2} = O(\Delta x). \end{aligned}$$

Later in the paper we will support this finding with an actual numerical experiment.

First order convergence rate would still be poor by modern standards. However, in view of the foregoing discussion it is clear that using an MLP representation of the flux gradient instead of the flux itself would suffice for the purpose of a second order accurate scheme. In fact, the primitive of the MLP representation, the flux function, would now have a valid Taylor expansion up to $p=2.5$ and, by the above arguments, would be able to fulfill the requirements of second order convergence rate. Suitably smooth functions for higher order schemes could be also built starting from representations of correspondingly higher derivatives, although it would require additional integration operations further complicating the overall scheme. Thus, in the ‘Application to heat transport’ section we stick to second order accurate schemes.

### Regularisation

The presence of noise in the data prevents direct calculation of the flux gradient (and higher derivatives). To improve the data quality we therefore first undertake a regularization procedure. Tikhonov’s method was found particularly effective for this purpose^48–52^. The method solves an optimization problem in which, given a set of noisy data $y_{i}({\mathbf{x}}_{i})$, the smoothed, noise reduced data, ${\hat{y}}$, is found by minimising an objective function, *Q*, containing two contrasting terms, one measuring ${\hat{y}}$’s fidelity to the original data and the other its smoothness. We evaluate the former term through the mean-squared-error (MSE) and the latter from high order derivatives ,$y^{(p)}$, as estimated from the regularised values, i.e.

8 $$\begin{aligned} Q((\hat{y}, y) = \Vert y - \hat{y}\Vert ^{2} + \lambda _{s} \sum _{p} \Vert \hat{y}^{(p)}\Vert ^{2}, \end{aligned}$$

where $\lambda _{s}$ is a parameter weighting the regularization term. Although Eq. (8) is typically formulated in the context of one-dimensional data, we apply it volumetrically, i.e. simultaneously to all different ${\mathbf{x}}$-space components. Thus ${\mathbf{x}}, y$ and $y^{(p)}$ correspond *linearised* one-dimensional data array. While increasing the computational complexity, this yields isotropic smoothness of $y({\mathbf{x}})$.

Using matrix operators

9 $$\begin{aligned} Q((\hat{y}, y) = (\hat{y}-y)^{\mathrm{T}}U(\hat{y}-y) + \lambda _{s} ({\mathcal{D}}^{p}\hat{y})^{\mathrm{T}}U({\mathcal{D}}^{p}\hat{y}). \end{aligned}$$

where U weights the element-wise contribution according to its volume measure in the MSE metric (i.e. the $d{\mathbf{x}}$ element in an integral) and ${\mathcal{D}}^{p}$ consists of a stack of partial differential operators with respect to all components of ${\mathbf{x}}$ including, in general, mixed terms up to order *p* (see ‘Regularisation’ in Methods for additional details). In the application example discussed in the next Section, where *y* is the heat flux and ${\mathbf{x}}$ the thermodynamic variables, we find it sufficient to include only non-mixed terms of order $p=4$. The optimal set, ${\hat{y}}$, is found by solving the equation obtained by setting $\partial Q/\partial {\hat{y}}$ to zero, hence

10 $$\begin{aligned} {\hat{y}} = \left( I + \lambda _{s} {\mathcal{D}}^{p^{\mathrm{T}}} U {\mathcal{D}}^{p}\right) {^{-1}} y. \end{aligned}$$

As for the $\lambda _{s}$ parameter, we follow the practice of setting its value such that the resulting $(y - {\hat{y}})$ statistics is close to what is expected for the random errors of the data. In particular, when the noise standard deviation, $\sigma _{y}$, is known, Morozov^52^ principle can be applied and

11 $$\begin{aligned} \lambda _{s} = \mathop {{{\,\mathrm{arg\,min}\,}}}\limits _{\lambda _{s}} \left( \sigma _{{\hat{y}}}(\lambda _{s}) -\sigma _{y}\right) ^{2}. \end{aligned}$$

When $\sigma _{y}$ is unknown, generalised cross-validation method^50^ can be applied instead. In our experiments we find these two methods to give virtually identical results and use Morozov’s for computational convenience.

### Latent representation

**Figure 3:**
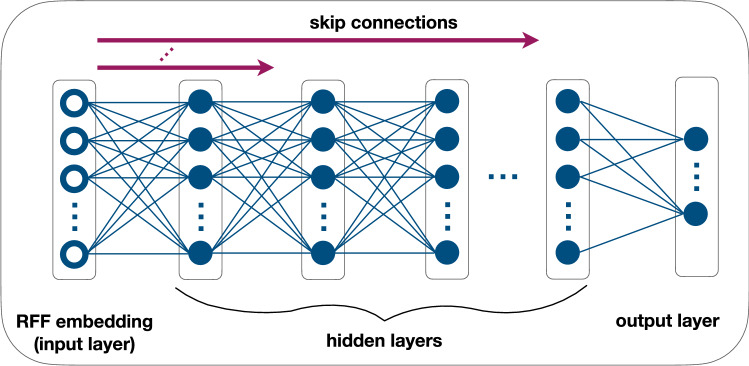
Trainable MLP representing the flux-gradient function. The first layer embeds the input features via Random Fourier Features (RFFs) which are then fed to the first hidden layer. The number of RFFs is the same as the number of hidden units which is constant across the hidden layers. Nonlinearity is introduced by application of a ReLU activation function to the affine mapping returned by the hidden units. The RFF embeddings are also fed to every other hidden layer except the last through skip connections. The output layer consists of as many regression units as the gradient components without activation function.

In order to obtain a latent representation of the transfer process of interest we use an MLP with the architecture summarised in Fig. 3 and described in detail in ‘MLP Architecture’ in Methods. The MLP is trained to learn the gradient of the flux function using a set of labels obtained by differentiating the regularised input flux data, as described in the previous section. The training is based on Stochastic Gradient Descent using an objective function given by the MSE of the relative error of the predicted value with respect to its label (further details are given in ‘MLP Architecture’ in Methods).

While in our application example discussed later on the flux density and its gradient depend on the point-wise values of the thermodynamic variables, a more general dependence from values in a neighbouring region of the evaluation point may at times be required. For such case an MLP may not suffice and a more flexible implementation of our machinery is conceivable, in which our MLP unit is embedded within a non-local-neural-network or even a full graph-network^30,53,54^ (of which the former is a special type).

## Application to heat transport

### Basics

We now consider the case of heat transport in a high temperature plasma, a case of realistic scientific and engineering interest. As already mentioned in the Introduction, classically this process is described by Fick’s law with a thermal diffusion coefficient given by Spitzer–Härm model^20^

12 $$\begin{aligned} q_{SH}&=-\kappa \, T_{e}/L_{T}, \quad \kappa = \frac{128}{3\pi } \zeta n_{e} v_{te} \lambda _{ei},\nonumber \\ v_{te}&= \left( \frac{2T_{e}}{m_{e}}\right) ^{\frac{1}{2}}, \; \lambda _{ei} =\frac{3T_{e}^{2}}{\sqrt{32\pi }Z^{2}n_{e} e^{4} \Lambda }, \; \zeta =\frac{0.24+Z}{4.2+Z}, \end{aligned}$$

where $T_{e},\, v_{te},\, n_{e},\, \lambda _{ei}$ are the electron temperature, thermal speed, number density and collisional mean free path, respectively, $L_{T} \equiv T_{e}/\nabla T_{e}$ is the temperature gradient scale-length, *Z* is the ions charge state, *e* the electric charge, $m_{e}$ the electron mass, and $\Lambda$ the Coulomb logarithm. The model equations (12), become invalid (and the heat flux strongly suppressed) as the electron mean free path approaches the temperature gradient scale-length, i.e. ^5–7^. Kinetic models based on a phase-space description of the plasma continue to apply so one possibility would be to learn the representation of latent heat flux function from such simulation data. For simplicity, however, in the following we use a dataset of values generated from the following heat flux function^11^

13 $$\begin{aligned} q(n_{e},T_{e},B)= n_{e}T_{e} v_\parallel = \epsilon \, n_{e}T_{e} v_{te}, \quad \epsilon (n_{e},T_{e},B)\equiv \frac{v_\parallel }{v_{te}}, \end{aligned}$$

where *B* is a magnetic field strength, $v_\parallel$ is the velocity component parallel to the magnetic field and

14 $$\begin{aligned} \epsilon (n_{e},T_{e},B) = \left( \frac{L_{T}}{\lambda _{ei}}+\beta _{e}+4\right) ^{-1}, \quad \beta _{e}=\frac{n_{e}T_{e}}{B^{2}/8\pi }. \end{aligned}$$

yielding a suppression factor

$$\begin{aligned} \epsilon \,L_{T}/\lambda _{ei} \end{aligned}$$

with respect to Spitzer–Härm’s flux, $q_{SH}=n_{e}T_{e}v_{te}\lambda _{ei}/L_{T}$.

Physically, this model describes the heat flux suppression due to whistler instabilities occurring in a low density, high-$\beta$ intergalactic plasma, characterised by $n_{e}\approx 10^{-4}$ cm$^{-3}$, $T_{e}\approx$ a few keV, and $B\approx 10^{-6}$ G, in the presence of temperature gradients with scales $L_{T}\approx 10^{22}$ cm^11^. In other words the above equations describe a specific emergent behavior of the heat flux suppression mechanism, caused by specific complex microscopic processes operating over several $\lambda _{ei}$ scales. As already pointed out, however, the heat flux suppression is a phenomenon occurring whenever irrespective of the underlying mechanism responsible for it. So for conditions relevant to, e.g., High Density Plasma Physics and Inertial Confinement Fusion, with similar keV temperatures and $n_{e} \approx 10^{19}$–$10^{23}$ cm$^{-3}$ but not necessarily supporting ordered magnetic fields, there would still be a transition to non-local transport for a temperature scale $L_{T}\approx 10^{-1}$–$10^{-5}$cm although the driving physical mechanism may differ^6,7^. Likewise can be said of plasmas characterised by different parameters that still combine to produce a similar value of $L_{T}T_{e}^{2}/n_{e}$. In each of these cases the cause and specific characteristics of the emergent behaviour will be different. However, our purpose here is to demonstrate that if such an behavior exists and can be described in terms of a set of input parameters characteristic of the plasma state, then we shall be able to capture it.

### Datasets

To build the various datasets for the supervised training we consider a domain defined by the parameter ranges in Table 1 and discretise it with a uniform grid. At each grid point, ${\mathbf{x}}=(n_{e},~ T_{e},~ \beta _{e})$, we then evaluate the flux function $y=q({\mathbf{x}})$ according to Eq. (13). To assess the impact of the data volume on the model’s performance we have generated three sets of data with different sampling density described by the number-of-points-per-decade parameter, $N_{ppd} = 5,~ 10,~ 20$. As detailed in the next Section the computed gradient function share the same grid as the flux function except for a surface layer one grid-point wide where the gradient cannot be fully computed. To compensate for this we pad the grid surface with a one grid-point wide buffer zone. The size of the buffer zone is then doubled to also account for an additional layer of gradient data that we do not use due to the degrading performance of the regularization step there. Thus, in general the grid dimensions are, $(N_{n}, N_{T}, N_\beta )=(2N_{ppd}+4, ~N_{ppd}+4, ~2N_{ppd}+4)$, with a corresponding grid spacing $\Delta \xi =(\xi _{max}-\xi _{min})/(N_{\xi }-1)$, with the min and max values given in the Full Grid section of Table 1, and $N_{\xi }$ the grid size corresponding the parameter $\xi$. Notice that because of the different grid spacings for different values of $N_{ppd}$ the actual parameter range covered by the gradient data differs for the different datasets, as illustrated by the ‘Gradient Grid PR-$N_{ppd}$’ sections in the lower part of Table 1. The flux suppression factor $\epsilon L_{T}/\lambda _{ei}$, also computed in the table, continues nonetheless to range from values $\ll 1$, i.e. the regime of highly suppressed flux, to values $\approx 1$, corresponding to the Spitzer–Härm limit. Finally, to assess the impact of noise in the data, we also generate datasets in which the flux-density values are modified to include a normally distributed random percentage error,

15 $$\begin{aligned} y\leftarrow & {} (1+\hat{\sigma }_{n}) y \end{aligned}$$

with $\hat{\sigma }_{n}$ a random variate from a Gaussian distribution ${\mathcal{N}}(0, \sigma _{n})$.

The list of datasets is summarised in Table 2. The first column is the name of the dataset and the second the value of $\sigma _{n}$ multiplied by 100. The next three columns indicate the $N_{ppd}$ parameter, the buffer size, and the total number of flux function evaluations, respectively. The last four columns refer to the flux gradient, in particular the total number of evaluations and the size of the training, validation and testsets partitions, respectively.

**Table 1:** Grid of parameter space: range and spacing for the grids of plasma parameter values at which the heat flux function (top) and its gradient (bottom three panels) are evaluated at different sampling density ($N_{ppd}$).

Parameter	Min	Max	Spacing
**Full grid**			
$n_{e}$ (cm$^{-3}$)	$10^{-5}$	$10^{-3}$	Uniform
$T_{e}$ (keV)	1	10	Uniform
$\beta _{e}$	$10^{-1}$	10	Uniform
$\epsilon \, L_{T}/\lambda _{ei}$	$7.0 \times 10^{-4}$	1.0	–
**Gradient grid PR-20**			
$n_{e}$ (cm$^{-3}$)	$5.6 \times 10^{-5}$	$9.5 \times 10^{-4}$	Uniform
$T_{e}$ (keV)	1.8	9.2	Uniform
$\beta _{e}$	$5.6 \times 10^{-1}$	9.5	Uniform
$\epsilon \, L_{T}/\lambda _{ei}$	$4.8 \times 10^{-3}$	0.9	–
**Gradient grid PR-10**			
$n_{e}$ (cm$^{-3}$)	$9.6\times 10^{-5}$	$9.1 \times 10^{-4}$	Uniform
$T_{e}$ (keV)	2.4	8.6	Uniform
$\beta _{e}$	$9.6 \times 10^{-1}$	9.1	Uniform
$\epsilon \, L_{T}/\lambda _{ei}$	$9.6 \times 10^{-3}$	0.8	–
**Gradient grid PR-5**			
$n_{e}$ (cm$^{-3}$)	$1.6 \times 10^{-4}$	$8.5 \times 10^{-4}$	Uniform
$T_{e}$ (keV)	3.3	7.8	Uniform
$\beta _{e}$	1.6	8.5	Uniform
$\epsilon \, L_{T}/\lambda _{ei}$	0.02	0.6	–

For each Table section, the last line shows the range of values of the heat-flux suppression factor. The temperature gradient length is fixed at $L_{T}=3\times 10^{22}$ cm.

**Table 2:** Datasets: the columns represent the datasets’ name, the percentage of random relative noise added to the flux function, the sampling density represented by the points-per-decade parameter, the total number of buffer grid points, and the total number of flux function evaluations.

Name	$\sigma _{n}$ ($\times 100$)	$N_{\mathrm{ppd}}$	$N_{\mathrm{buf}}$	$N_{q}$	$N_{\nabla q}$			
					Total	Training	Eval.	Test
A.0	0	20	4	46,464	32,000	21,760	5440	4800
A.1	1	20	4	46,464	32,000	21,760	5440	4800
A.5	5	20	4	46,464	32,000	21,760	5440	4800
A.10	10	20	4	46,464	32,000	21,760	5440	4800
A.20	20	20	4	46,464	32,000	21,760	5440	4800
B.0	0	10	4	8064	4000	2720	680	600
B.1	1	10	4	8064	4000	2720	680	600
B.5	5	10	4	8064	4000	2720	680	600
B.10	10	10	4	8064	4000	2720	680	600
B.20	20	10	4	8064	4000	2720	680	600
C.1	1	5	4	1764	500	340	85	75
C.0	0	5	4	1764	500	340	85	75
C.5	5	5	4	1764	500	340	85	75
C.10	10	5	4	1764	500	340	85	75
C.20	20	5	4	1764	500	340	85	75

The last four columns relate to the gradient function datasets (three components each), including the total number of evaluations and its partitions into training (68%), evaluation (17%) and test (15%) sets, respectively.

#### Data regularization

For each dataset in Table 2 we apply Tikhonov’s regularization to the log values of *y* as a function of the log values of *x*. This means that our fidelity term in Eq. (8) is a relative error. The regularization term is given by the 4-th derivative computed on the regularised data based on finite differences of adjacent cell values (we effectively apply the operator ${{\mathcal{D}}}^{4,1,*}_{N_{n}, N_{T}, N_{\beta }}$ described in ‘Regularization’ in Methods). The flux gradient, providing the labels for the MLP’s supervised learning discussed above, is then computed using a second-order accurate central difference scheme (the operator ${\mathcal{D}}^{1,2,*}_{N_{n}, N_{T}, N_\beta }$) on the regularised data. Thus, for the $\xi$ component

16 $$\begin{aligned} y^{(1)}_{\xi }({\mathbf{x}}_{i}) = \frac{{\hat{y}}({\mathbf{x}}_{i}+\Delta \xi \, {\hat{{\varvec{\xi }}}}) - {\hat{y}}({\mathbf{x}}_{i}-\Delta \xi \, {\hat{{\varvec{\xi }}}})}{2\Delta \xi }. \end{aligned}$$

**Figure 4:**
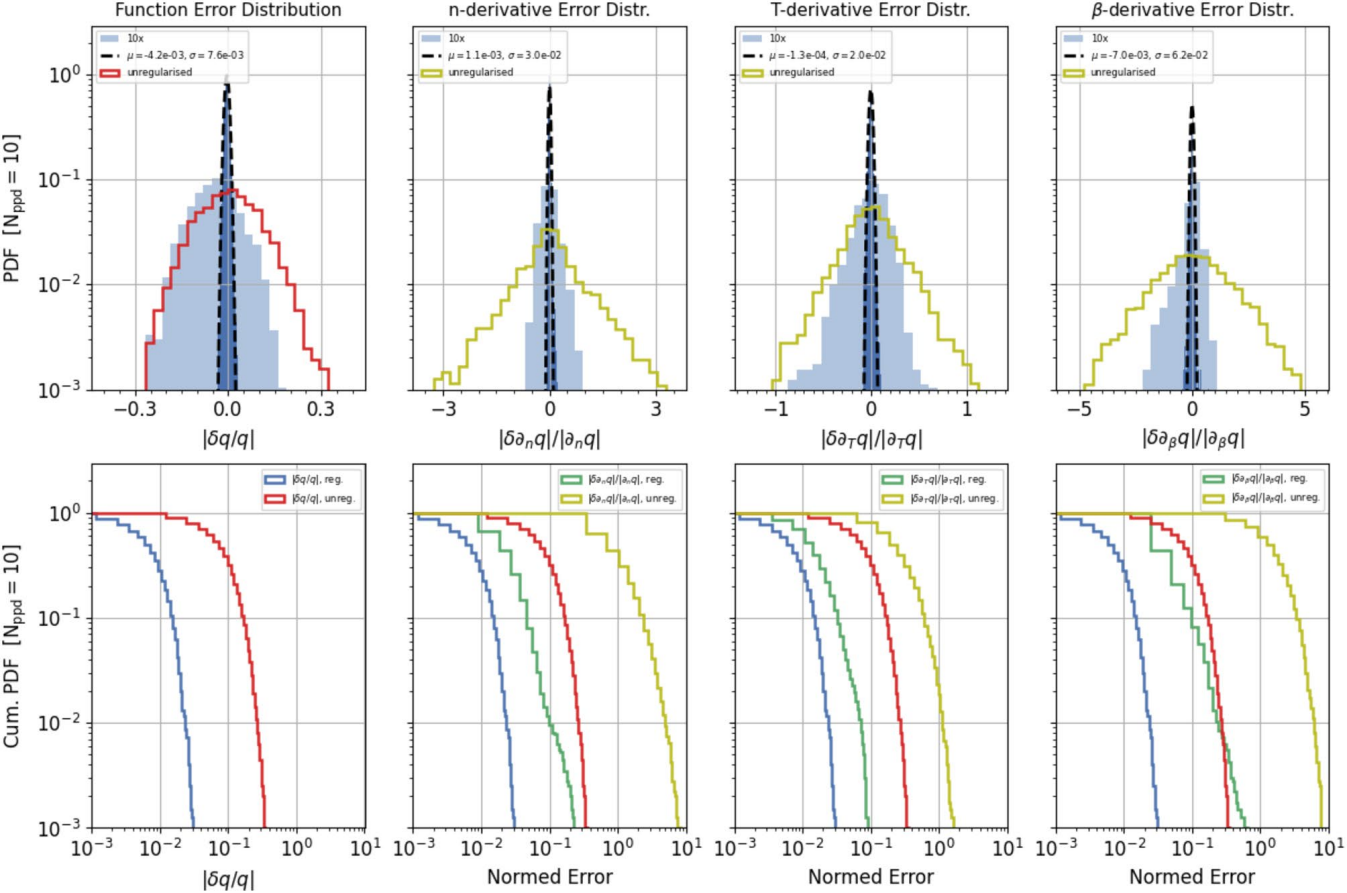
Single regularization result: regularization results for the dataset B.10, with $N_{ppd}=10$ and $\sigma _{n}=0.1$. Top panels: histograms of the relative error distribution of the regularised and unregularised flux function (left) and each gradient component (next three panels), respectively (see legend for details). The blue shaded regions correspond to the regularised error distribution expanded by a factor 10. Bottom panels: corresponding cumulative error distributions for the histograms in the top panels, particularly the regularised and unregularised flux function data (blue and red) and its individual gradient components (green and olive), respectively.

Figure 4 shows the results of the regularisation procedure in terms of the accuracy of the flux function and its gradient component, for the specific case of the dataset B.10, i.e. $N_{ppd}=10$ and $\sigma _{n}=0.1$. The top panels from left to right show the relative error distribution of the regularised and unregularised flux and of each of its gradient component, respectively. The blue shade corresponds to the regularised error distribution expanded by a factor 10. The bottom part similarly compares, in the corresponding panels, the cumulative error distributions of the regularised and unregularised flux function (blue and red) and its gradient components (green and olive), respectively. The plot shows that Tikhonov’s regularization is very effective at suppressing the noise in the original data allowing reliable estimates of the function gradients even in the case of relatively high noise. In this particular case we effectively obtain errors RMS below 1% and of order of a few % for the flux function and its gradient components, respectively, starting from a dataset with 10% normally distributed relative error. The improvement is particularly dramatic for the flux gradient whose calculation, as is well known, would be otherwise very challenging.

**Figure 5:**
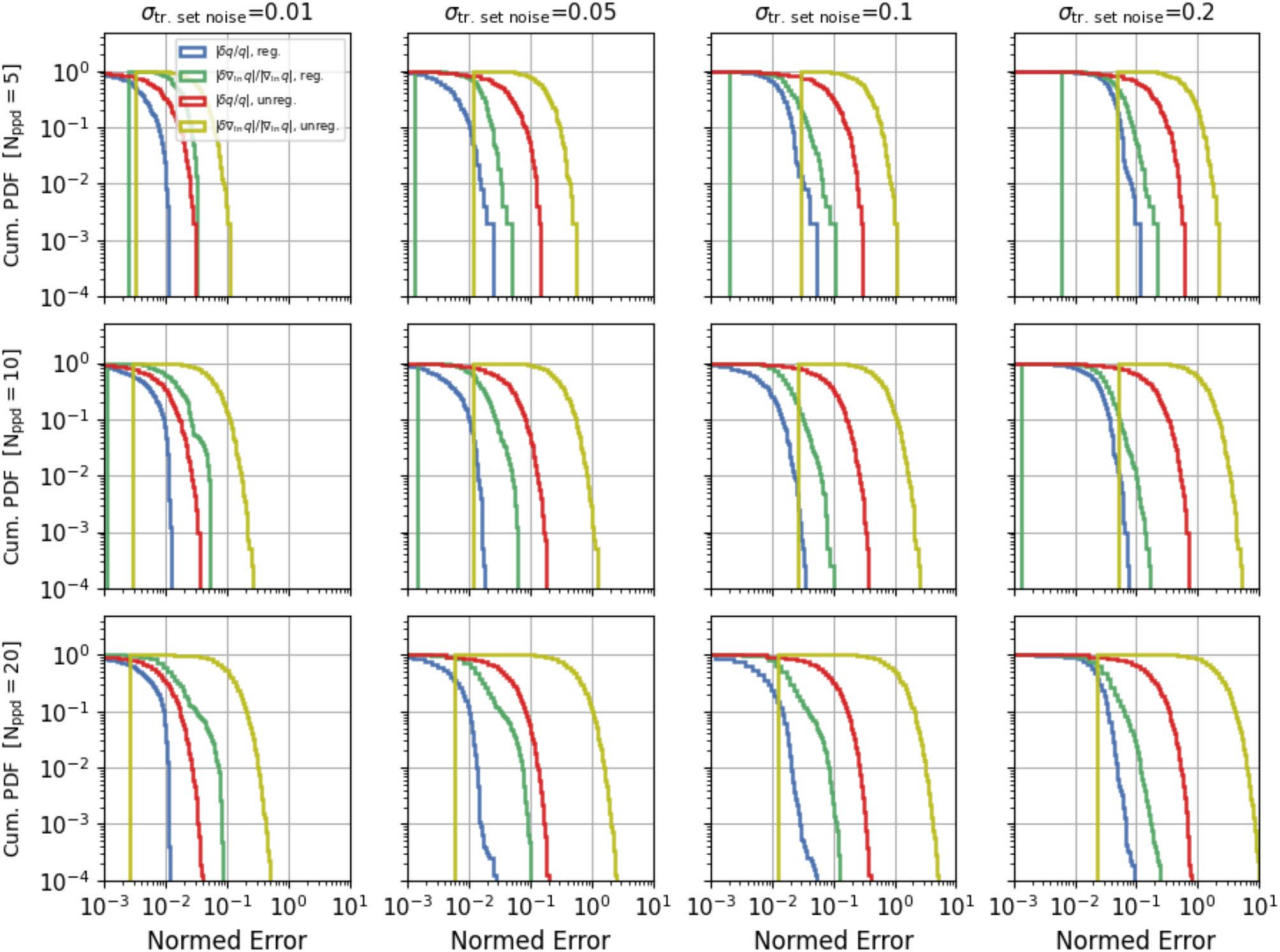
Regularization results: for each combination of the $N_{ppd}$ and $\sigma _{n}$ parameters, the blue and red curve show the cumulative distributions of the relative error of the flux function while the green and olive curves show the cumulative distributions of the flux gradient relative error, estimated by the ratio of the Euclidean norm of the flux log-gradient error and the Euclidean norm of the correct flux log-gradient (see main text for definition of log-gradient and Euclidean norm).

The results for all datasets are summarised in Fig. 5. For each combination of the $N_{ppd}$ and $\sigma _{n}$ parameters, the various panels show the cumulative distribution of the relative error of the flux function and its gradient, for the the regularised (blue and green) and unregularised (red and olive) data, respectively. To estimate the flux gradient error, we compute the Euclidean norm of the flux log-gradient error and divide it by the Euclidean norm of the correct flux log-gradient. Here log-gradient of $f({\mathbf{x}})$, with ${\mathbf{x}}$ a vector, means that $(\nabla _{\log {\mathbf{x}}}f)_{i}=\nabla _{\log x_{i}}f$ and Euclidean norm of ${\mathbf{x}}$ means $(\sum _{i} x_{i}^{2})^{1/2}$. Additional statistics on the errors and their trends with the $N_{ppd}$ parameter are shown in Supplementary Fig. S2. Consistently with Figs. 4, and 5 and Supplementary Fig. S2 show the effectiveness of the regularization, which allows us to compute estimates of the flux gradient with an accuracy that in most cases is significantly better than even that of the original data. It is tempting but somewhat not straightforward to compare the panels in Fig. 5 not corresponding to the same $N_{ppd}$. In fact, on the one hand as already pointed out the domain of the regularised data differs for different values of the $N_{ppd}$ parameter. On the other hand, the higher $N_{ppd}$, i.e. the grid resolution, the higher the impact of noise on the calculation of the gradient components, as is clearly visible in Supplementary Fig. S2. In the following we will show that it is definitely advantageous to use finer grids, i.e., larger $N_{ppd}$.

#### Training data

The MLP is trained on a set of data consisting of features, ${\mathbf{x}}$, the thermodynamic variables already discussed, and labels $\mathbf{y}^{(1)}$ corresponding to the gradient of the flux function obtained from the regularised data. We take the natural logarithm of both except for the $\beta$ component of the flux gradient for which we take the log of the negative value, and normalise the resulting ${\mathbf{x}}$ and ${\mathbf{y}}^{(1)}$ components to have zero mean and unit standard deviation.

### Learned representations

For each dataset listed in Table 2 we train a total of 100 MLP-models with hyperparameters selected from a (reduced) search space given in Table 5 (see ‘Hyperparameter Optimization’ in Methods for further details). Table 3 shows a selection of best models with the corresponding used dataset, hyperparameters and also final RMS and Max *evaluation* errors. We aimed for evaluation errors to be at least consistent with those characterising the regularised data. We have repeated the analysis and tests shown in this section with alternative selection of best models obtained during the hyperparameter search and found consistent results.

**Table 3:** Best models selection: from left to right the columns include the model’s name, the name of training set as listed in Table 2, the number of hidden layers and units, respectively, the $\sigma _{RFF}$ parameter for generation of Random Fourier Features embeddings, and final the RMS and Max statistics for the *evaluation* errors.

Model	Dataset	Learning rate	Neural network			Regularization		Result	
			Layers	Units	$\sigma _{\mathrm{RFF}}$	Type	Param.	RMS error	Max error
**A-series:** ${\mathbf{N}}_{\mathbf{ppd}}$** = 20**									
MA.0	A.0	$1.23\times 10^{-3}$	4	1024	0.963	L1	$1.02\times 10^{-6}$	$7.6\times 10^{-3}$	$3.9\times 10^{-2}$
MA.1	A.1	$1.80\times 10^{-3}$	4	512	0.733	–	–	$7.5\times 10^{-3}$	$3.4\times 10^{-2}$
MA.5	A.5	$3.42\times 10^{-3}$	5	256	0.601	L1	$6.45\times 10^{-6}$	$1.1\times 10^{-2}$	$9.4\times 10^{-2}$
MA.10	A.10	$1.05\times 10^{-3}$	6	512	0.663	–	–	$8.8\times 10^{-3}$	$6.5\times 10^{-2}$
MA.20	A.20	$2.53\times 10^{-3}$	6	1024	1.723	L1	$8.44\times 10^{-7}$	$1.7\times 10^{-2}$	$3.1\times 10^{-1}$
**B-series:** ${\mathbf{N}}_{\mathbf{ppd}}$** = 10**									
MB.0	B.0	$1.13\times 10^{-3}$	4	512	0.790	L2	$6.49\times 10^{-3}$	$8.7\times 10^{-3}$	$5.6\times 10^{-2}$
MB.1	B.1	$2.88\times 10^{-3}$	6	512	0.739	–	–	$9.9\times 10^{-3}$	$6.3\times 10^{-2}$
MB.5	B.5	$1.57\times 10^{-3}$	5	512	0.564	L2	$8.25\times 10^{-4}$	$9.0\times 10^{-3}$	$8.0\times 10^{-2}$
MB.10	B.10	$2.15\times 10^{-3}$	4	512	0.965	L2	$1.01\times 10^{-4}$	$9.4\times 10^{-3}$	$8.1\times 10^{-2}$
MB.20	B.20	$2.66\times 10^{-3}$	6	256	0.844	L1	$9.79\times 10^{-6}$	$1.0\times 10^{-2}$	$1.0\times 10^{-1}$
**C-series:** ${\mathbf{N}}_{\mathbf{ppd}}$** = 5**									
MC.0	C.0	$1.30\times 10^{-3}$	5	1024	0.444	L1	$6.18\times 10^{-7}$	$2.4\times 10^{-2}$	$8.2\times 10^{-2}$
MC.1	C.1	$3.34\times 10^{-3}$	5	128	0.515	–	–	$2.4\times 10^{-2}$	$1.3\times 10^{-1}$
MC.5	C.5	$1.89\times 10^{-3}$	6	256	0.545	–	–	$2.5\times 10^{-2}$	$2.5\times 10^{-1}$
MC.10	C.10	$2.03\times 10^{-3}$	5	128	0.483	L1	$6.82\times 10^{-6}$	$2.7\times 10^{-2}$	$1.9\times 10^{-1}$
MC.20	C.20	$2.51\times 10^{-3}$	5	128	0.547	L2	$1.00\times 10^{-2}$	$2.7\times 10^{-2}$	$1.8\times 10^{-1}$

The table is divided into three subtables, one for each density of sampling parameter, $N_{ppd}$, characterising the training data.

In the following we compute various statistics of the model prediction errors in which all gradient components are treated without distinction. This is feasible because the models are trained to learn the log of the labels, and the prediction errors correspond to relative errors (hence the axis label). It also means that the computed error refer equally to each component.

**Figure 6:**
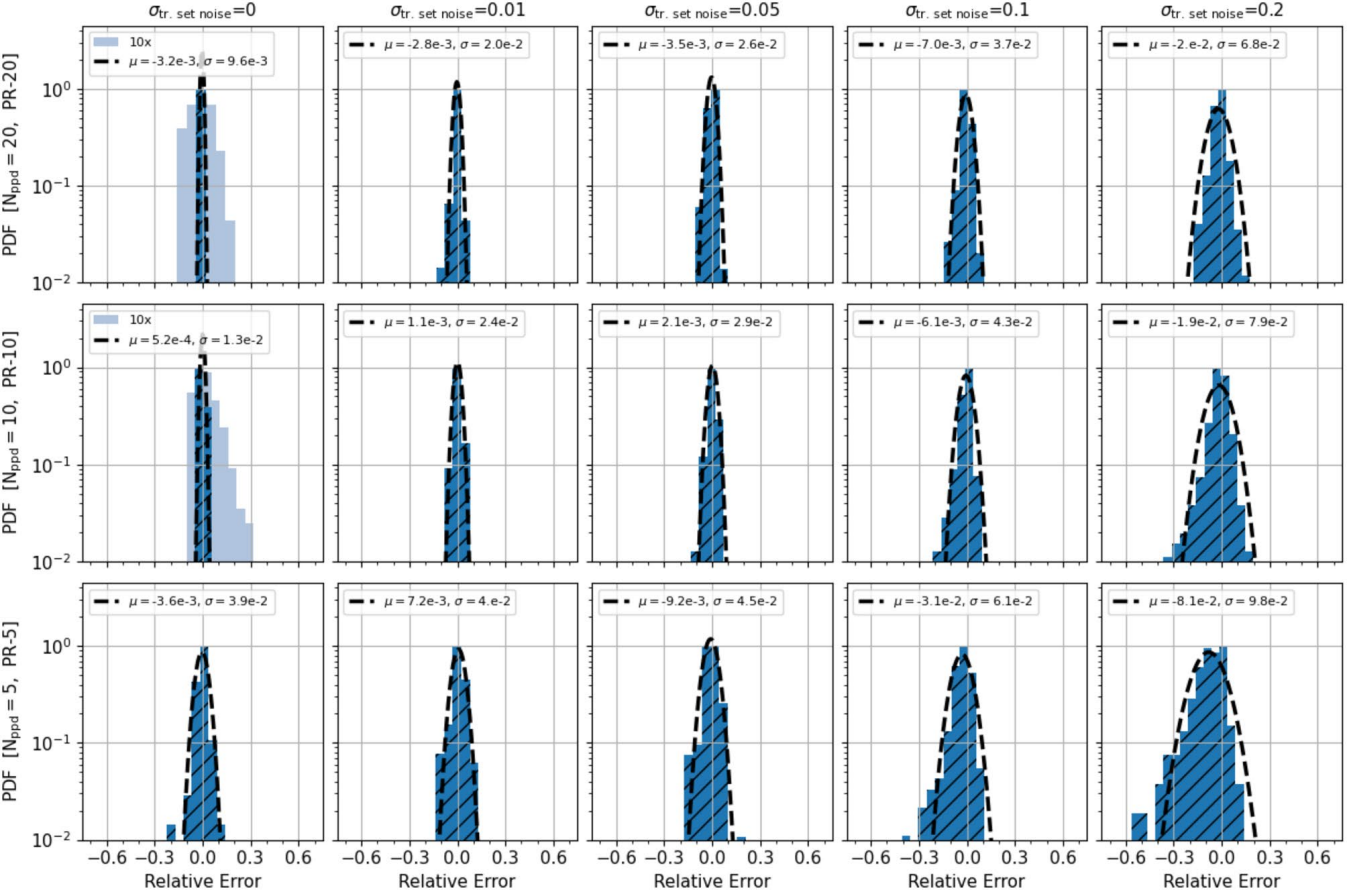
MLP models test errors: Histogram of the *test-errors* of the MLP models in Table 3. All the histograms are rescaled so that they all peak at 1. Each row corresponds to a different $N_{ppd}$ (and parameter range domain PR-$N_{ppd}$), while each column corresponds to a different value of $\sigma _{n}$, the noise in the flux dataset before applying Tikhonov’s regularization. The errors are computed with respect to the noiseless testsets, i.e. the A.0, B.0 and C.0 testsets for models of the A-, B-, C-series, respectively. The legend shows the mean $\mu$ and standard deviation $\sigma$ of the histogram in each panel. The lightblue shapes in some panels correspond to a histogram of 10$\times$ larger errors.

Figure 6 shows a summary of the *test-errors* for the models in Table 3. For each model of the A-, B-, C- series the errors are computed with respect to the noiseless testset, i.e. the testsets of the A.0, B.0 and C.0 datasets respectively. Each row corresponds to a different $N_{ppd}$ (and parameter range domain PR-$N_{ppd}$), while each column corresponds to a different value of $\sigma _{n}$, the noise in the flux dataset before applying Tikhonov’s regularization. The bias $\mu$ is typically negligible with respect to the variance term and, as it would be expected, in general the performance improves consistently for smaller values of the noise, $\sigma _{n}$, and for denser datasets, i.e. larger $N_{ppd}$. It is interesting to note that for large values of the input noise, i.e. $\sigma _{n} =10, 20$, the MLP representations are characterised by an RMS error per component $\simeq \sigma _{n} /2 \simeq 2 \sigma _{reg}$, i.e. half the pre-regularization error but twice as large that of the regularised data (Supplementary Fig. S2). This is likely a consequence of the fact that the residual error noise in the regularised data is not purely Gaussian and the MSE is a less effective objective for closing in on the ground truth. Here, we also see that although in Supplementary Fig. S2 the error values for the regularised data at given percentile appear to plateau, MLP representations trained with more densely sampled datasets always shows an overall better performance. Below we show a more direct comparison of the models tested within the same parameter domain. In any case it is remarkable that we can obtain representations of the flux gradient with errors per components of only a few percent, despite an input relative error of the flux data of even 10%.

**Figure 7:**
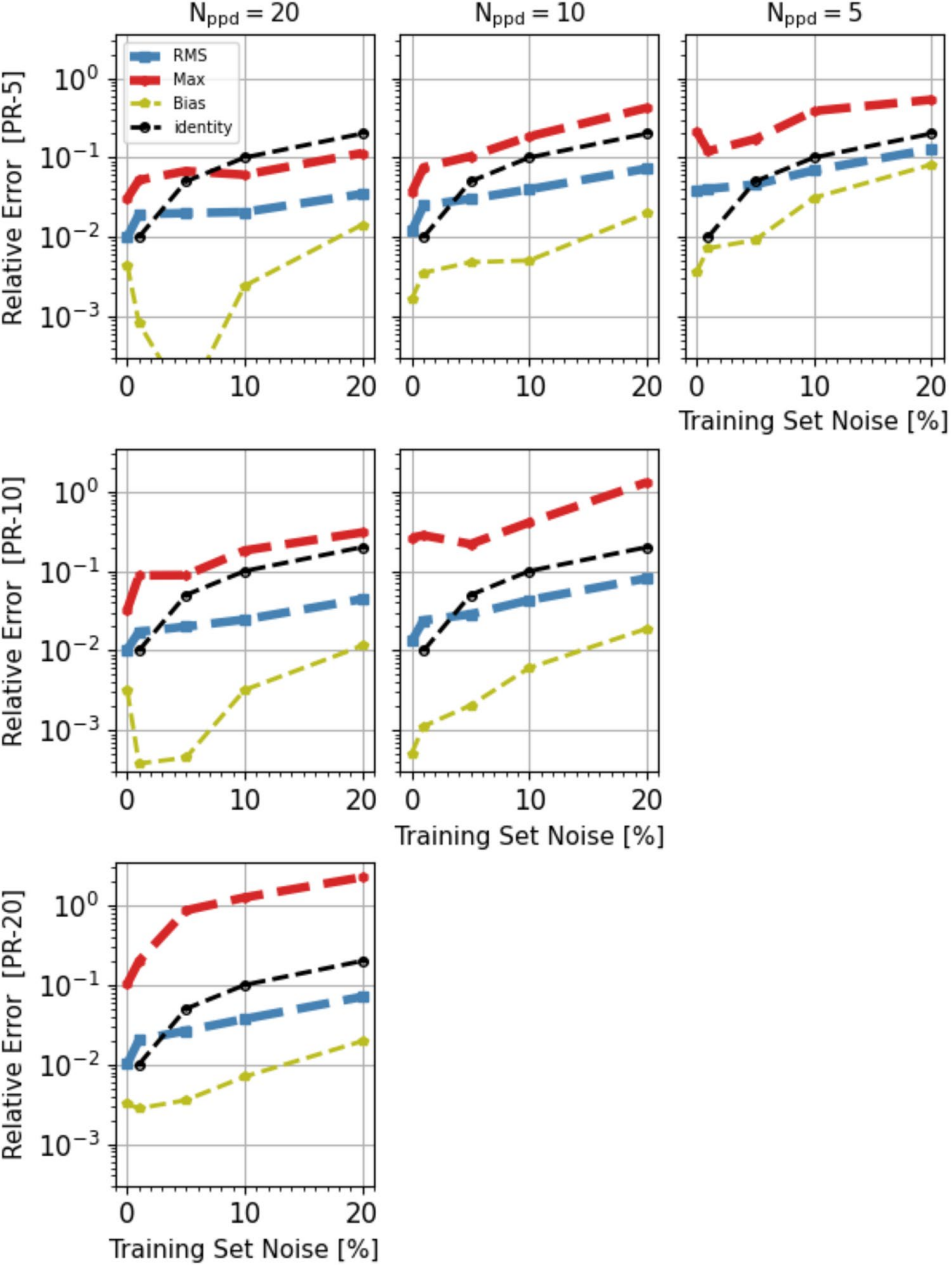
Test-error Statistics: RMS (blue dash line), Max (red dash line) and Bias (yellow thin dash line) statistics of the model prediction errors and their trend with the pre-regularization noise for different sampling size cases. In particular, from top to bottom, the rows correspond to errors computed using the noiseless testset of the C-, B- and A-Series respectively, while from left to right the columns correspond to models trained with datasets with $N_{ppd}=20, 10$ and 5, respectively. The half-filled points threaded by the black dash line correspond to the case of equal relative error and input noise (the identity line, which would be diagonal in a linear plot).

Figure 7 shows more specific error statistics, in particular the RMS (blue dash line), Max (red dash line) and Bias values (yellow thin dash line), characterising the model predictions and their trend with the pre-regularization noise. From top to bottom, the rows correspond to errors computed using the noiseless testset of the C-, B- and A-series respectively, while from left to right the columns correspond to models trained with datasets with $N_{ppd}=20, 10$ and 5, respectively. The half-filled points threaded by the black dash line correspond to the case of equal relative error and input noise (the identity line, which would be diagonal in a linear plot).

**Figure 8:**
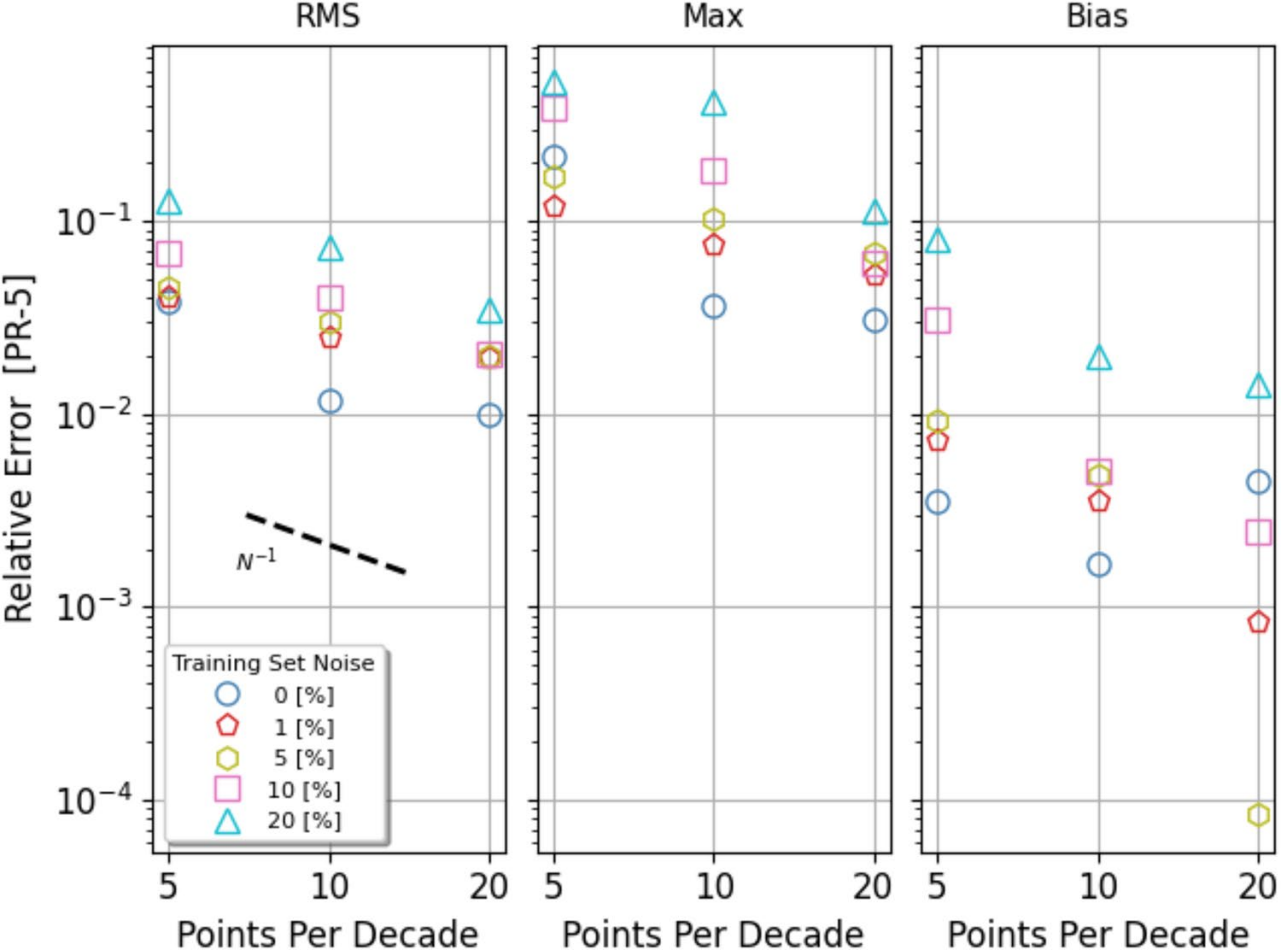
Trend with data sampling density: Test-errors’ RMS (left), Max (center) and Mean (left) presented in the top three panels of Fig. 7, relative to the PR-5 domain, plotted as a function of $N_{ppd}$. Different symbols correspond to models trained with data characterised by different pre-regularization noise (see Figure’s legend). The error statistics appear to roughly decrease as the inverse of the parameter $N_{ppd}$ (black dashed line).

In general the error statistics appear to trace the value of the pre-regularization noise. The RMS value remains well below the identity line, consistent with the quality of the gradient data obtained after Tikhonov’s regularisation, except at the low noise end, $\sigma _{n}\le 10^{-2}$. Since panels on the same row correspond to prediction errors computed using the same testset, their comparison shows that models trained with larger datasets, i.e. with larger $N_{ppd}$ are significantly more accurate. This is shown more specifically in Fig. 8 where the statistics presented in the top three panels of Fig. 7, relative to the PR-5 domain, are plotted as a function of $N_{ppd}$ in three separate panels. Although the data points are not aligned along straight lines, there appear to be a trend for the various statistics to decrease linearly with the parameter $N_{ppd}$. Related to the improvement of the model performance as we move along the rows of Fig. 7 from right to left, which is attributed to a higher sampling density of training data, there is a corresponding performance worsening as we compare panels from top to bottom along vertical columns which, in addition to the reverse of the above effect, includes the additional difficulty of modeling the function’s gradient on a progressively larger domain.

### Convergence tests

To test the performance of our MLP representation in a numerical context, we set out to compute the temperature evolution of a plasma in a one-dimensional domain. The thermodynamic state of the plasma is constant in space, except for a sinusoidal perturbation with 5% amplitude in both the temperature and a second randomly chosen variable (*n* or *T*). The temperature evolution is computed by time integrating the Eq. (1), using a second order accurate numerical scheme for hyperbolic equations^55^ that employs the heat flux gradient provided by our MLPs. The algorithm, further detailed in ‘Numerics’ in Methods, is a higher order extension of Godunov method using a predictor corrector scheme. The computational domain has periodic boundary conditions and is discretised with $N_{\mathrm{Mesh} \,\mathrm{Points}}$ resolution elements. The performance is based on the convergence rate of the numerical solution, i.e. the rate at which the error drops as a function of $N_{\mathrm{Mesh} \,\mathrm{Points}}$. The numerical error is computed using Richardson’s extrapolation method (also further detailed in ‘Numerics’ in Methods). We repeat the numerical integration test for a sample of 30 runs with different, randomly chosen, unperturbed ($n, T, \beta$) plasma parameters.

**Figure 9:**
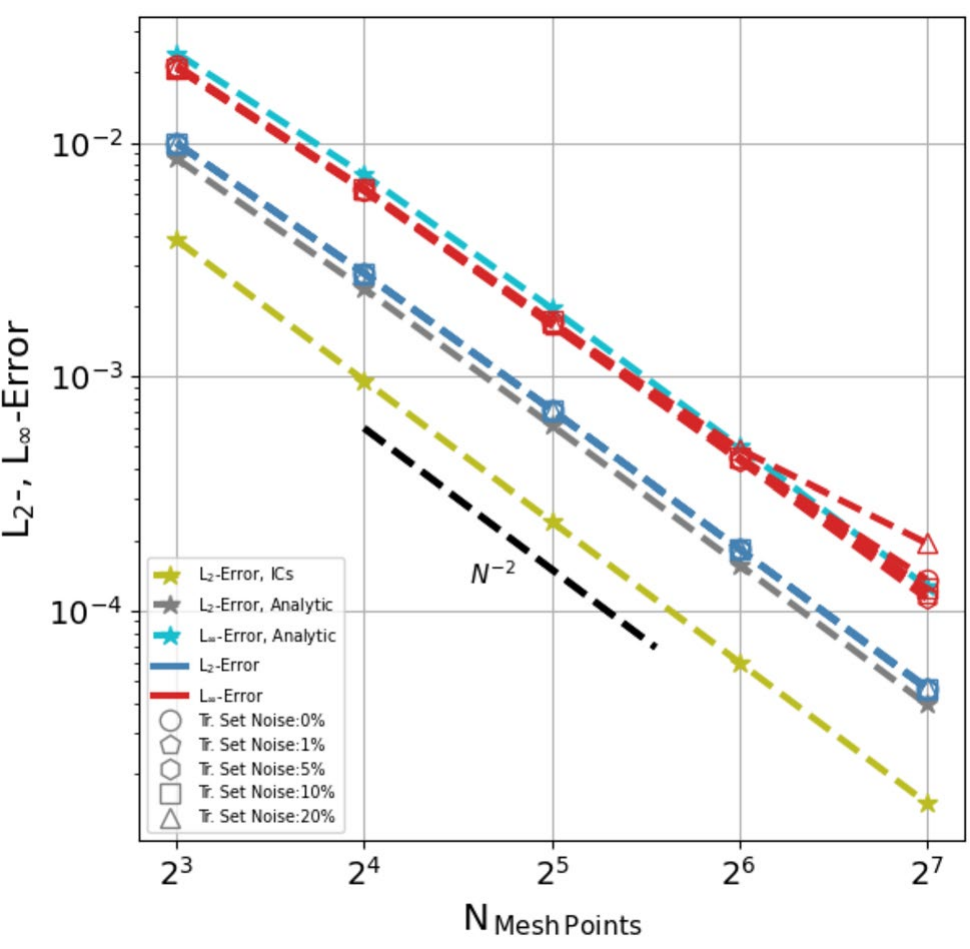
Convergence test: $L_{2}$ and $L_{{\mathrm{inf}}}$ error norms for the implementations using an MLP model of the flux gradient (blue and red, respectively) and for the implementation using instead an analytic expression (gray and cyan, respectively, with the cyan curve multiplied by 1.15 to make it visible). The plotted errors are averages over a sample of 30 runs using different, randomly chosen, unperturbed values of the thermodynamic parameters, ($n, T, \beta$). Models trained with data characterised by different pre-regularization noise are represented by different symbol (see legend), though they are difficult to distinguish as their calculated error data points mostly overlap. The $L_{2}$ error norm is also shown for the initial conditions (olive). Expected error drop rate for a second order accurate scheme is indicated by the black dash line.

The results are summarised in Fig. 9 where, for each MLP model, labeled according to the noise characterising the unregularised heat-flux data, the sample averaged $L_{2}$ (blue dashed line) and $L_{{\mathrm{inf}}}$ (red dashed line) error norms are plotted as a function of, $N_{\mathrm{Mesh~ Points}}$. The corresponding results for the analytic form of the heat-flux function are also shown (gray and cyan dashed line for $L_{2}$ and $L_{{\mathrm{inf}}}$ respectively) together the $L_{2}$ error of the initial conditions (olive dashed line), providing a simple sanity check. The $L_{2}$ and $L_{{\mathrm{inf}}}$ errors norms of the various MLP based integration scheme implementations actually overlap and, more importantly, display a second order convergence rate (see black line), as expected for a second order accurate scheme. The slight flaring of $L_{{\mathrm{inf}}}$ at the high resolution end is sensitive to the sinusoidal amplitude and is perhaps indicative of nonlinear effects. The implementation using the analytic heat-flux function shows the same convergence—actually this test simply verifies the correctness of our code implementation—as would any other analytic expression, including the results of our regression analysis. Notice that the numerical solutions corresponding to each implementation do converge to different results. This is a consequence of the consistency property of the scheme^3^. In fact, in each case we are modeling a slightly different equation specified by the specific flux function model (analytic, MLP or symbolic regression) utilised in the numerical scheme.

The second order convergence of the implemented scheme confirms the error analysis presented earlier in Eq. (7). To further illustrate that it is the stochastic oscillation of the gradient in the neighbor of an expansion point that causes the random error cancellation of the spurious term and the resulting partial improvement of the convergence rate with respect to the expectation from the truncation error, we carry out the following simple experiment. We implement the above integration scheme using the flux model in Eq. (6) and compare the results obtained with three different models for *a*: in addition to the original ‘stochastic’ value sampled between [-1, 1] we also set $a=0$ (i.e. the ‘analytic’ model) and $a=1$ (‘fixed’ value). We carry out the same numerical integration tests as above except that we now perturb only the temperature component. The results for the three models are shown in Supplementary Fig. S2 together with the usual ICs sanity check. The plot shows that while the ‘stochastic’ model (gray) displays second order convergence rate (see bottom black line) as the the ‘analytic’ model (blue), the ‘fixed’ model (red) converges only as $N^{-1.5}$. This would be expected for a consistent error build up due to the spurious fixed term during each time-step integration.

### Symbolic regression

In this final stage we attempt to recover a mathematical expression for the heat flux function through a symbolic regression analysis. For this purpose we use the Deep Symbolic Optimisation package^56^ which builds the mathematical expressions through a recurrent neural network trained with a reinforcement learning method. We first, however, precondition our symbolic regression in two ways: first, as suggested in^46^, we restrict the search to expressions with sensible physical dimensions. In addition, we exploit knowledge of the asymptotic limit of the sought mathematical expression if known. This is common practice when seeking to extend a law of physics to previously unexplored regimes (e.g., from classic to the relativistic or quantum limits). Both of these measures help reduce the symbolic search space which grows exponentially with the number of components. As already mentioned, the heat flux is well known when the electron mean free path is small compared to the temperature gradient scale, i.e.

17 $$\begin{aligned} \lim _{\lambda _{ei}/L_{T} \rightarrow 0} q(n_{e},T_{e},B_{e}) = q_* \,\frac{\lambda _{ei}}{L_{T}}, \quad q_*=n_{e}T_{e}v_{te}. \end{aligned}$$

Since $q_*$ has the same physical dimensions as *q*, we only need to search for a multiplicative dimensionless factor, our $\epsilon$ in Eq. (13), which will be a function of dimensionless variables. Given the dimensional physical quantities entering our plasma physics problem, $(n_{e},T_{e},B_{e},m_{e},e,L_{T})$, only three dimensionless combinations are possible (or combinations thereof) namely, $L_{T} n_{e} e^{4} T_{e}^{-2} \propto L_{T}/\lambda _{ei} \equiv x_{1}$, which in fact already appears in the asymptotic limit (17), $n_{e}T_{e}/B^{2} \propto \beta _{e} \equiv x_{2}$, and, $n_{e}^{1/3}L_{T}\equiv x_{3}$, which actually $\epsilon$ does not depend upon. Notice that from the point of view of the symbolic regression there is no advantage in choosing $x_{2}=\beta _{e}$ or $x_{2}= n_{e}T_{e}/B^{2}$, or $x_{1}$ versus $3x_{1}$ for that matter, because the analysis will have to figure out the value of those coefficients by itself. Instead, we chose $\beta _{e}$ and $L_{T}/\lambda _{ei}$ because they have a clear physical meaning.

The datasets for the symbolic regression consist of 2000 entries containing the values of the target function, $\epsilon \equiv q/q_*$ and the corresponding independent variables $(x_{1}, x_{2}, x_{3})$. The entries are randomly sampled from the four Datasets A.1, A.5 A.10 and A.20 in Table 2, allowing us to compare the performance of the symbolic regression under various conditions of data quality. Our function set includes a minimal choice of $\{+, -,\times , \div , \mathrm{const.} \}$, with a max of three constants, as well as the functions $\log$ and $\exp$, allowing for generic exponential expressions, often seen in physics, like, ‘$\exp (g(\log (x))$’, where *g* is an arbitrary combination of the function set. We avoid trigonometric functions, which are not expected in this problem. We set $\mathtt{batch\_{s}ize}=10^{4}$ and $\mathtt{n\_{s}amples}=10^{6}$, resulting in 100 iterations and use standard settings otherwise. The chosen number of iterations appears sufficient for the reinforcement learning’s reward function to reach a plateau, but is otherwise arbitrary and longer runs could lead to slightly better accuracy. The DSO optimizes an objective function given by the Normalised Root Mean Squared Error (i.e. the root of the MSE of the relative error) of the function prediction, similar to the case of the previously discussed regularisation and MLP training.

The results are summarised in Table 4 where for each dataset we report the symbolic expression obtained through the regression, the RMS and Max of the relative error for both the flux and its gradient, computed on a random sample of 10$^{6}$ entries. The symbolic regression appears to successfully retrieve the correct functional form of the factor $\epsilon$, clearly outperforming the MLP model in terms of uncertainties of the flux gradient. Exception is made for the case of the A.5 Dataset, in which the symbolic formula is found to be characterised by an unusually large error. This result is rather peculiar and illustrates the importance of running the regression with multiple random initialisation seeds to obtain statistically robust results. The other interesting observation is that there seems to be no obvious advantage from running the DSO on the regularised data. As in the case of the MLP models, this seems related to the non Gaussian character of the residual error in the regularised data, which the NRMSE minimization carried out by the DSO is not effective at reducing. In this respect it is interesting to compare the statistics of residual errors after Tikhonov’s regularization given in Supplementary Fig. S2 and those of the symbolic regression in Table 4, and notice, with a grain of salt, their comparable magnitude.

**Table 4:** Results from the symbolic regression analysis.

Dataset	Symbolic expression for $\epsilon ^{-1}$	Function error		Gradient error	
		RMS	Max	RMS	Max
**Function set = {+, −, ×, ÷, log, exp, const., Iterations = 100**					
A.1	$(0.9992\cdot x_{1} + 1.0008\cdot x_{2} + 3.9928)^{1.00055}$	$7.3\times 10^{-4}$	$1.9\times 10^{-3}$	$1.6\times 10^{-3}$	$3.9\times 10^{-3}$
A.5	$(1.0977 \cdot x_{1} + x_{2} + 3.8453919)/\log (0.3062\cdot \log (x_{3}))$	$7.3\times 10^{-2}$	$1.6\times 10^{-1}$	$1.3\times 10^{-1}$	$2.5\times 10^{-1}$
A.10	$(x_{1} + x_{2} + 3.9388) \cdot e^{0.0013\cdot x_{2}}$	$8.3\times 10^{-3}$	$1.5\times 10^{-2}$	$1.3\times 10^{-2}$	$1.4\times 10^{-1}$
A.20	$(0.9855\cdot x_{1} + 1.0145\cdot x_{2} + 3.8580)^{1.0108}$	$1.5\times 10^{-2}$	$3.7\times 10^{-2}$	$3.1\times 10^{-2}$	$7.6\times 10^{-2}$

From left to right the column indicate the name of the dataset from which the training data were sampled, the found symbolic expression corresponding for clarity to $\epsilon ^{-1}$, the RMS and Max statistics of the relative error for the function and its gradient on a random sample of 10$^{6}$ entries.

In conclusion, it is difficult to predict the performance of the DSO in the case of more complex functional dependencies between features and labels, and when the statistics of the data errors is not purely Gaussian. Nevertheless, the performance shown in a study of realistic research interest even with high levels of data noise is encouraging, particularly from the perspective of using experimental data.

## Conclusions

In this paper we use a ML based approach to improve basic knowledge, mathematical description and numerical modeling capability of generic transport processes. Ours is part of ongoing efforts to employ modern artificial intelligence techniques in science and overlaps in scope with developments of augmented schemes and accelerators for numerical simulations, as well as methods to gain insight in and possibly reach discovery of new laws of physics through symbolic regression analysis. Transport processes may be ruled by complex micro-physics which is impractical to model theoretically, but may exhibit emergent behavior describable by a closed mathematical expression. We are, therefore, particularly interested in formulating transport terms, *q*, employable in a continuum mechanics macroscopic description, by learning from data provided either by microscopic-scale numerical simulations, progressively closer to first principles, or even directly from experiments, for those physical conditions under which even current ab initio codes do not provide consistent results.

Ideally one would be able to learn the transport term, *q*, as a mathematical expression obtained through a symbolic regression analysis. This is the preferred path because it allows for easier implementation in computational modeling and is very valuable to theoretical analysis. However, it is also the less certain path, because it is an intrinsically more difficult task due to the unknown complexity of the sought relation and, amongst others, the degrading impact of the data uncertainties on its performance^46^. Alternatively, the transport term, *q*, can be expressed via an MLP representation. Care must be taken, however, with its implementation in numerical integration codes. Due to its noisy character, its Taylor expansion is unreliable beyond the first order term, leading to truncation errors $\tau (\Delta x) \sim O(\Delta x^{1/2})$. We have nevertheless shown that owing, in particular, to the peculiar stochastic character of the error term spoiling the Taylor expansion, using an MLP representation of $\nabla q$ instead of *q* itself, allows us to effectively recover a flux function sufficiently smooth for implementation in a second order accurate code. Formulations for even higher order schemes are feasible but add complexity and were not explicitly pursued here. In any case, in this approach it becomes necessary to first regularize the flux data, to minimize the impact of the error noise on the calculation of the flux gradients, which provide the labels for our trainable MLP function. Our method of choice for this purpose is Tikhonov’s.

When applied to an idealised study of heat transport relevant to astrophysical and thermonuclear fusion plasmas we find that Tikhonov’s regularization is very effective at cleaning the data from Gaussian noise, allowing accurate estimates of the flux gradient. The MLP’s trained on such labels deliver in general flux gradient representations of relatively high quality, with their overall performance that, while reflecting the pre-regularization noise level, appears to improve roughly linearly with the density of parameter sampling (Fig. 8). For example, for a relative error noise of 10% and $N_{ppd}=20$ our MLP representation computes the flux gradient components with an error RMS of 3.7% (Fig. 6). Interestingly, the MLP training based on regularised data does not lead to model prediction errors that are further reduced with respect to the error characterising the training data. This is clear when comparing the error RMS of the regularised gradient data in Supplementary Fig. S2 and of the MLP gradient predictions in Fig. 6. In particular, for each MLP representation, at the low-end of the $\sigma _{n}$ values we have a prediction error RMS $\simeq \sigma _{n}$ while at the high-end, i.e. $\sigma _{n} =10, 20$, we have a prediction error RMS $\simeq \sigma _{n} /2 \simeq 2 \sigma _{reg}$, i.e. half the pre-regularizaton noise but twice as large the error of the regularised data (Supplementary Fig. S2). We believe this is caused by the non Gaussian character of the residual error of the regularised data which makes the MSE a less effective objective for closing in on the ground truth. Finally, the latent representation defined by our MLPs is shown to be suitable for implementation in second order accurate schemes and can be used in computational models of the continuity equation as a plug-in to augment the numerical integration algorithm and deploy and accurate description of the transport process of interest.

The application of the DSO symbolic regression package to our idealised study of heat transport, complemented by some preconditioning operations of the problem, leads to a successful results mostly outperforming the precision of the MLP model, though it may be useful to run the regression with multiple random initializations to help avoiding occasional lack of performance. In line with the findings related to the MLP results, we observe that running the DSO on the regularised data appears to produce little or no benefit. This seems again related to the non Gaussian character of the regularised data’s error which the DSO optimization based on the NRMSE is not so effective at reducing. In this respect, we note the comparable performance of Tikhonov’s regularization in Supplementary Fig. S2 and the DSO prediction in Table 4 in terms of error statistic.

The foregoing discussion suggests that while it is preferable to have accurate data it is also important to have control over the error statistics in order to employ the most appropriate objective function. Along the same line, we expect our results to remain valid beyond the specific case of random Gaussian relative error assumed in our idealised study, provided the objective functions are modified accordingly.

In conclusion, the successful retrieval of accurate MLP and symbolic representations of the heat flux function in the context of a study that is somewhat idealised, yet representative of the field^11^, appears a promising first step to be able to employ in macroscopic models the necessary sophisticated information on transport processes implicitly available from microscopic descriptions. The robustness of the results even in the case of significant noise in the data is also very attractive, particularly in view of applications using experimental data.

## Methods

### MLP architecture

The architecture of our MLP is summarised in Fig. 3. At its core is a number, $N_{Layers}$, of hidden layers each with the same number, $N_{Units}$, of hidden units, both tunable parameters. We embed the input features into a set of Random Fourier Features^57,58^ (RFF), i.e. given the input vector $\mathbf{x}$, we define the components

18 $$\begin{aligned} \mathbf{x}_{i} \leftarrow \cos ({\mathbf{k}}_{i}{\cdot {\mathbf{x}} + \phi }_{i}), i\in \{i: 0\le N_{\mathrm{RFF}} \}. \end{aligned}$$

with $N_{\mathrm{RFF}}=N_{Units}$. As in^58^ we observe no benefit when training the parameters ${\mathbf{k}}_{i}$ and $\phi _{i}$, so following^57^ we randomly sample the $\mathbf{k}_{i}$’s from the distribution, ${\mathcal{N}}(0,\sigma _{\mathrm{RFF}})$, with $\sigma _{\mathrm{RFF}}$ a tunable parameter, and the $\phi _{i}$’s uniformly in the interval $[0, 2\pi )$. A ReLU activation function is applied to the affine mapping returned by the hidden units. We also employ skip connections to feed the RFF embeddings to every other hidden layer except the last. The output layer consists of as many regression units as the gradient component without activation function. To train the MLP we define a loss function given by the Mean Squared Error of the predicted value with respect to the label. To prevent overfitting we early stop the training if the accuracy does not improve during a number of consecutive iterations given by a patience parameter set to 100.

### MLP representation of the flux function

The MLP representation of the flux function uses the architecture described in the previous section with the following custom choice of the otherwise tunable parameters: 5 hidden layers each consisting of 512 units, a $\sigma _{\mathrm{RFF}}=0.95$ for RFF embedding and a $L_{2}$ regularization with parameter $\lambda =10^{-5}$. The MLP was trained with the noiseless data of the A.0 set using a learning rate of $10^{-3}$. It reached convergence after 410 integration steps resulting in an RMS error of $9.6\times 10^{-3}$ and a MAX error of $3.8\times 10^{-2}$.

### Regularization

In this section we develop the tools to compute *f*’s derivative of order *p*, $f^{(p)}$, based on finite difference schemes. This are the tools employed for the calculation of the regularization term in Tikhonov’s method.

We consider a *D*-dimensional parameter space discretized by a grid $\Xi \in {\mathbb{R}}^{D}$ of dimensions $n_{0},\dots n_{D-1}$ and a scalar function $f:{\varvec{\xi }} \in \Xi \longrightarrow {\mathbb{R}}$. The grid elements, ${\varvec{\xi }}$, identified by the set of indexes $i_{0}, i_{1}, \dots i_{D-1}$, are not necessarily uniformly spaced but their parameter values grow monotonically with the respective index. In view of what will follow we define the utility function

$$\begin{aligned} {\mathcal{V}}_{\mathtt{cmp}, \,d} \equiv {\left\{ \begin{array}{ll} \Pi _{i \in A {:}{=}\{i \;\mid \; 0\le i < D \,\wedge \, i \,{\mathtt{cmp}} \,d\}} \; n_{i}, &{}\quad {\hbox {if }}\; A \ne \varnothing \\ 1 &{}\quad {\mathrm{otherwise}} \end{array}\right. } \end{aligned}$$

which takes as input a comparison operator, $\mathtt{cmp}$, and a dimension, *d*, and returns the volume of the subspace consisting of the dimensions fulfilling the comparison. For example, ${\mathcal{V}}_{>,\, 1}$ returns $n_{2}\times n_{3}\times \dots n_{D-1}$. We also define a stacking function

$$\begin{aligned} {{{\mathcal{S}}}} ({\mathcal{A}}^{q}, i, n)\equiv \begin{pmatrix} {\mathcal{A}}^{i}, {\mathcal{A}}^{i+1}, \cdots , {\mathcal{A}}^{i+n-1} \end{pmatrix} ^{T} \end{aligned}$$

that takes an indexed operator, ${\mathcal{A}}^{q}$, an initial index value, *i*, and a count, *n*, and returns a stack of *n* contiguously indexed operators starting from index *i*. In order to proceed we now linearise the grid of parameter values in $\Xi$ by arranging them into a 1-dimensional array, ${\mathcal{X}}$, according to an order in which the highest index runs fastest and the lower indexes progressively slower. Likewise we define a 1-dimensional array, ${\mathbf{y}}$, whose *i*-th element is $y_{i}=f(\mathbf{x}_{i})$, with ${\mathbf{x}}_{i}$ the *i*-th element of ${\mathcal{X}}$.

We can now define the matrix operator, $\Delta ^{1,\,m,\,d}_{n_{0},\ldots n_{D-1}} :{\mathbb{R}}^{n_{0}\cdot n_{1}\ldots n_{D-1}} \longrightarrow {\mathbb{R}}^{n_{0}\cdot n_{1}\ldots (n_{d}-m)\ldots n_{D-1}}$, computing the difference between values corresponding to grid points separated by *m* points along the *d* axis,whereThe partial differential operator with respect to the *d*-th component of ${\mathbf{x}}$ is then

$$\begin{aligned} {\mathcal{D}}^{1,\,m,\,d}_{n_{0},\dots n_{D-1}} = {\texttt {Diag}}^{-1}\left( \Delta ^{1,\,m,\,d}_{n_{0},\dots n_{D-1}} \cdot {{\mathbf{x}}}\right) \cdot \Delta ^{1,\,m,\,d}_{n_{0},\dots n_{D-1}} \end{aligned}$$

and

$$\begin{aligned} {\mathbf{y}}^{(1)} = {\mathcal{D}}^{1,\,m,\,d}_{n_{0},\dots n_{D-1}} \cdot {\mathbf{y}} = \frac{\Delta ^{1,\,m,\,d}_{n_{0},\dots n_{D-1}}\cdot {\mathbf{y}}}{\Delta ^{1,\,m,\,d}_{n_{0},\dots n_{D-1}} \cdot {\mathbf{x}}}, \end{aligned}$$

with the fraction in the last term meant component-wise. Likewise we can define the *p*-th order finite difference operator with respect to the *d*-th component of ${\mathbf{x}}$,

$$\begin{aligned} {\Delta }^{p,\, m, \, d}_{n_{0},\dots n_{D-1}} = \overbrace{ {\Delta }^{1,\, m, \, d}_{n_{0},\dots n_{d}-m*(p-1), \dots n_{D-1}} \cdot {\Delta }^{1,\, m, \, d}_{n_{0},\dots n_{d}-m*(p-2), \dots n_{D-1}} \cdot \dots {\Delta }^{1,\, m, \, d}_{n_{0},\dots n_{D-1}}}^{p \;{\mathrm{times}}} \end{aligned}$$

and the corresponding *p*-th partial differential operator

$$\begin{aligned} {\mathcal{D}}^{p,\,m,\,d}_{n_{0},\dots n_{D-1}} = {\texttt {Diag}}^{-1}\left( \Delta ^{p,\,m,\,d}_{n_{0},\dots n_{D-1}} \cdot {{\mathbf{x}}}\right) \cdot \Delta ^{p,\,m,\,d}_{n_{0},\dots n_{D-1}} \end{aligned}$$

so that

$$\begin{aligned} {\mathbf{y}}^{(p)} = {\mathcal{D}}^{p,\,m,\,d}_{n_{0},\dots n_{D-1}} {\mathbf{y}} = \frac{\Delta ^{p,\,m,\,d}_{n_{0},\dots n_{D-1}} \cdot \mathbf{y}}{\Delta ^{p,\,m,\,d}_{n_{0},\dots n_{D-1}} \cdot {\mathbf{x}}}. \end{aligned}$$

For $m=1$, the above operators map grid point values to midpoint interface values and for $m=2$ it maps grid point values to interior midpoint grid values. If *m* is even and the grid spacing is uniform, ${\mathbf{y}}^{(p)}$ and ${\mathbf{x, y}}$ share the same $\Xi$ grid except for an outer layer of thickness *m/2*, as finite differences cannot be computed normal to the boundary unless proper boundary conditions are provided. The above differentials can also be composed to build mixed differentiation. Finally, differential operators of various order and compositions can be stacked to define an overall matrix operators providing the regularization term in Tikhonov’s method. For example, a list of all partial derivatives of order *p* (excluding the mixed terms) is obtained by applying to the input vector ${\mathbf{y}}$ the operator obtained after stacking the individual ${\mathcal{D}}^{p,\,m,\,d}_{n_{0},\dots n_{D-1}}$ for $d=0,1,\dots D-1$, as follows

$$\begin{aligned} {\mathcal{D}}^{p,\,m,\,*}_{n_{0},\dots n_{D-1}} = {\mathcal S}\left( {\mathcal{D}}^{p,\,m}_{n_{0},\dots n_{D-1}}, 0, \,n_{D}\right) . \end{aligned}$$

### Numerics

Our numerical integration scheme is a simplified version of the predictor corrector scheme proposed by van Leer’s^55^. It is described in the pseudocode Algorithm 1, where $u=(n, T, \beta )^{\mathrm{T}}$ denotes the set of thermodynamic variables, $\Delta x$ and $\Delta t$ are the mesh and time-step size, $N_{x}$ and $N_{t}$ the numbers of meshes and integrations time-steps, respectively, P.B.C. stands for application of *periodic boundary conditions* necessary to complete the operations in the next code block. In addition JF is formally the Jacobian of the vector function whose components describe the flux of each thermodynamic variable. Since in this case only the heat-flux *q* is non-zero we have

$$\begin{aligned} \mathrm{JF}:u&\longrightarrow \begin{pmatrix} 0 &{}\quad 0 &{}\quad 0 \\ \partial _{n} q(u) &{}\quad \partial _{T} q(u) &{}\quad \partial _\beta q(u) \\ 0 &{}\quad 0 &{}\quad 0 \end{pmatrix}. \end{aligned}$$

where $\partial _{z}$ denotes the partial derivative with respect to *z*. Note that we do not use slope limiters.

The experiments reported in the ‘Convergence Tests’ are characterised by the following setup:

$$\begin{aligned} N_{x}&=\left\{ 2^{3}, 2^{4}, 2^{5}, 2^{6}, 2^{7}, 2^{8} \right\} \\ \Delta x&=\frac{1}{N_{x}}, \\ \Delta t&=\mathrm{CFL}\,\frac{\Delta x}{\lambda _{max}}, \\ N_{t}&= 2 \,\frac{N_{x}}{2^{3}}. \end{aligned}$$

with CFL = 0.5 and $\lambda _{max}$ the max value over the domain of the (only) eigenvalue of the problem, namely $\lambda =\partial _{T} q$, thus enforcing the CFL condition on the timestep.

The errors are measured using Richardson’s extrapolation. So, given the numerical result $T_{r}$ at a given resolution *r* we first estimate the error at a given grid point *i*, as

$$\begin{aligned} \varepsilon _{r, i} = T_{r,i} - {\bar{T}}_{r+1,i}, \end{aligned}$$

where ${\bar{T}}_{r+1}$ is the solution at the next finer resolution, spatially averaged onto the coarser grid (which is second order accurate). We then take the 2-norm and max-norm of the error,

$$\begin{aligned} L_{2}&= \Vert \varepsilon _{r} \Vert _{2} = \left( \sum |\varepsilon _{r,i}|^{2} v_{i}\right) ^{1/2},\\ L_{\infty }&= \Vert \varepsilon _{r} \Vert _\infty = \max (|\varepsilon _{r,i}|) \end{aligned}$$

where $v_{i}=\Delta x$ is the cell volume.
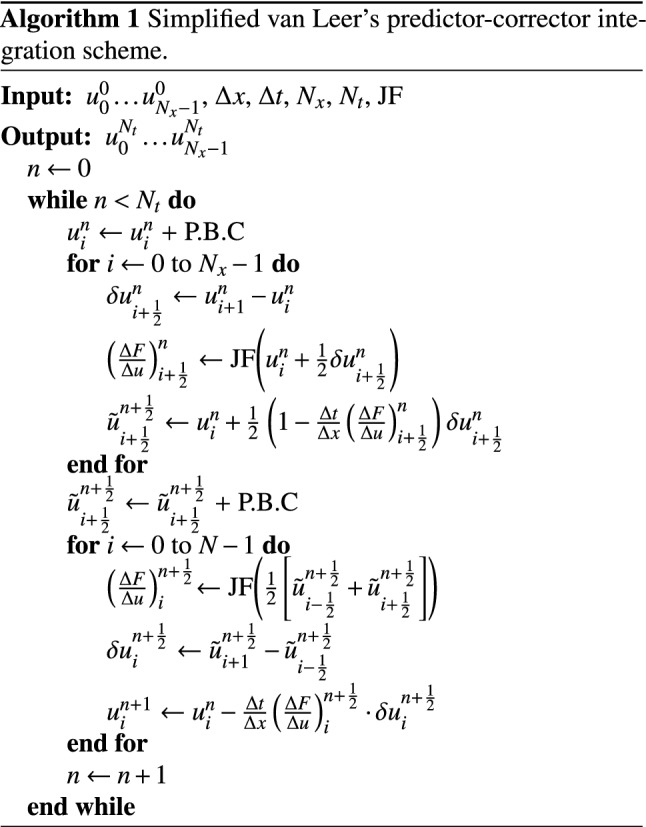


### Hyperparameter optimization

Our model is characterised by a number of hyperparameters, particularly the number of hidden layers and hidden units, the value of $\sigma _{\mathrm{RFF}}$, the initial value of the learning rate. Appropriate range of values for these parameters have become clear during the development and testing stages. In the final stage we perform additional optimal tuning by comparing for each dataset listed in Table 2 a total of 100 models with hyperparameters selected from the reduced search space given in Table 5. Other parameters not listed there include the batch size, typically set to 700, and the number of steps before the decay rate of the learning rate enters into effect, ranging between 800 and 1600. The hyperparameter optimisation is efficiently carried out with the orchestrator Ray Tune^59^.

**Table 5:** Reduced space searched for the final tuning of hyperparameters characterising the MLP model.

Hyperparameter	Search space	Space type
Number hidden layers	{4, 5, 6}	exhaustive
Number hidden units	{128, 256, 512, 1024}	exhaustive
Learning rate	[$10^{-4}$, 2$\times 10^{-3}$]	log-uniform sampling
$\sigma _{\mathrm{RFF}}$	[0.1, 5.0]	log-uniform sampling

### Implementation details

Our code is implemented in JAX^60^ and uses public libraries for both data-structures and algorithms. In particular, Tikhonov’s regularisation code makes extensive use of SciPy libraries for sparse matrix operations, while our Deep Learning code is based on libraries from Deepmind including Haiku^61^ for the MLP and Optax^62^ for the optimiser. The latter consists of a chain object combining an Adam algorithm^63^ with standard settings and a custom exponential-decay scheduler characterised by a drop rate of 0.9997 and a floor value of $10^{-5}$. The scheduler kicks in after an input number of steps varying between 800 and 1600.

## Supplementary Information

Supplementary Information.

## Data Availability

The datasets used and/or analysed during the current study available from the corresponding author on reasonable request.
