# Supplementary material for: Learning transport processes with machine intelligence
Source: Sci Rep. 2022 Jul 9;12:11709. doi: 10.1038/s41598-022-15416-y (PMC9271097; doi:10.1038/s41598-022-15416-y)
Supplement: Supplementary file 1 — Supplementary Information. [file 41598_2022_15416_MOESM1_ESM.pdf]

Supplementary materials

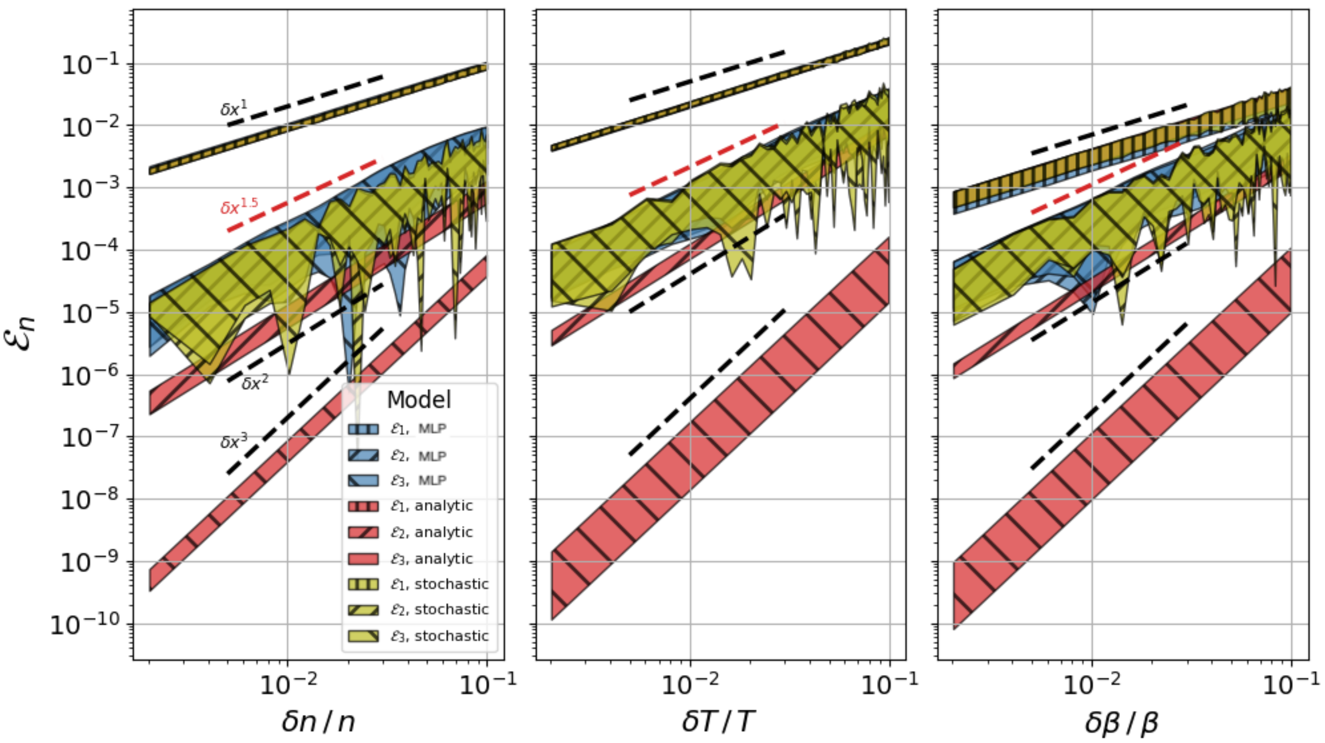

Figure S1. Taylor expansion's error II: same as Fig. 1 but with superposed data for the function in equation (6), the stochastic model, consisting of the analytic flux function q and a random term proportional to its first derivative multiplied by a number randomly sampled within $[-1,\ 1]$.

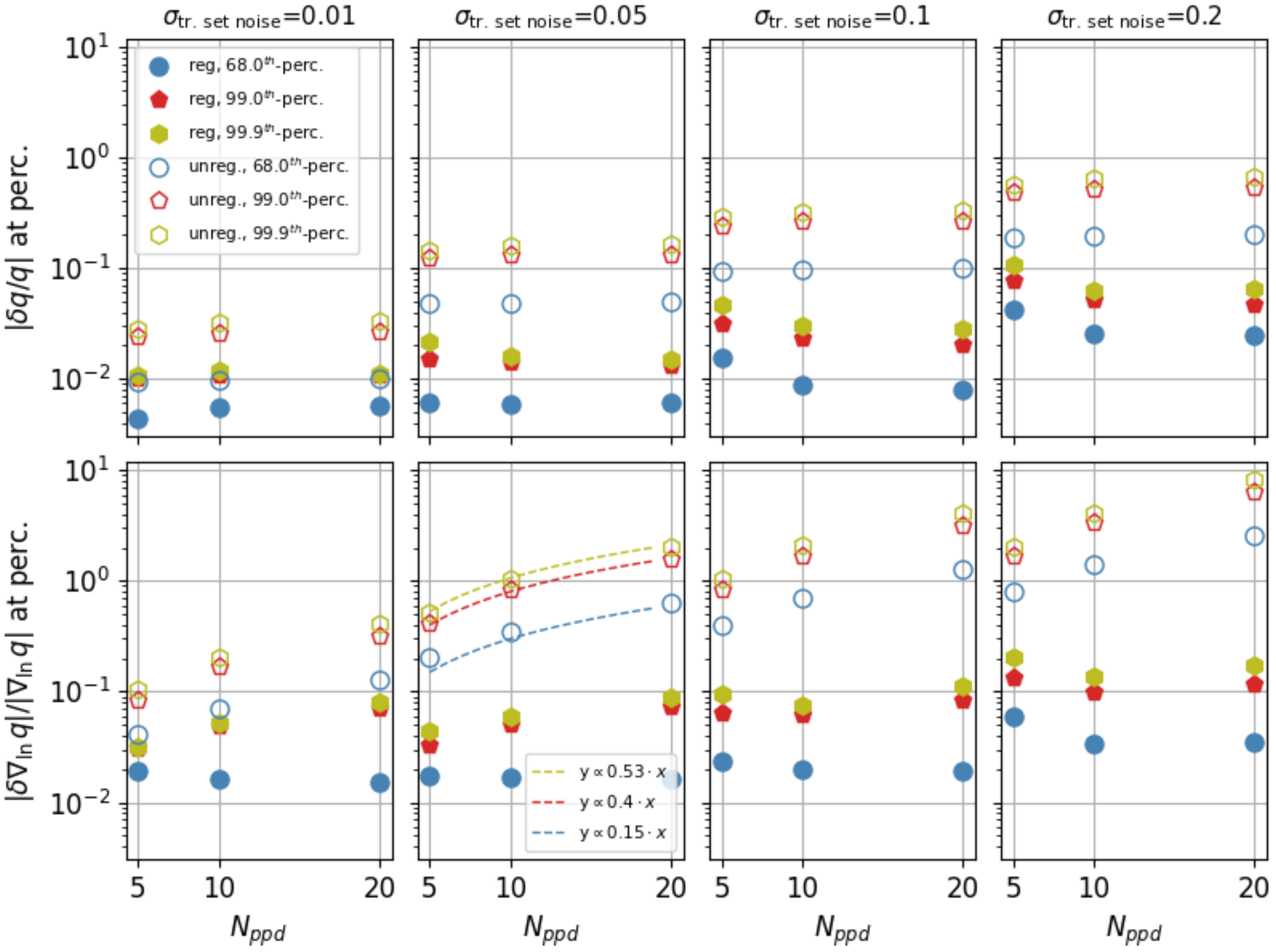

Figure S2. Errors at percentile: for each value of the pre-regularization noise, σ_n, the panels show as a function of sampling density represented by the (N_{ppd}) parameter, the relative error of the flux function (top) and the flux gradient (bottom), the latter estimated as the ratio of the Euclidean norm of the flux log-gradient error and the Euclidean norm of the correct flux log-gradient (see main text for definition of log-gradient and Euclidean norm), at 68th (blue circles), 99th (red pentagons), and 99.9th (olive hexagons) percentile, respectively. Filled and open symbols refer to regularised and unregularised data, respectively. The dashed lines in the second bottom panel from the left is a simple eyeball fit.

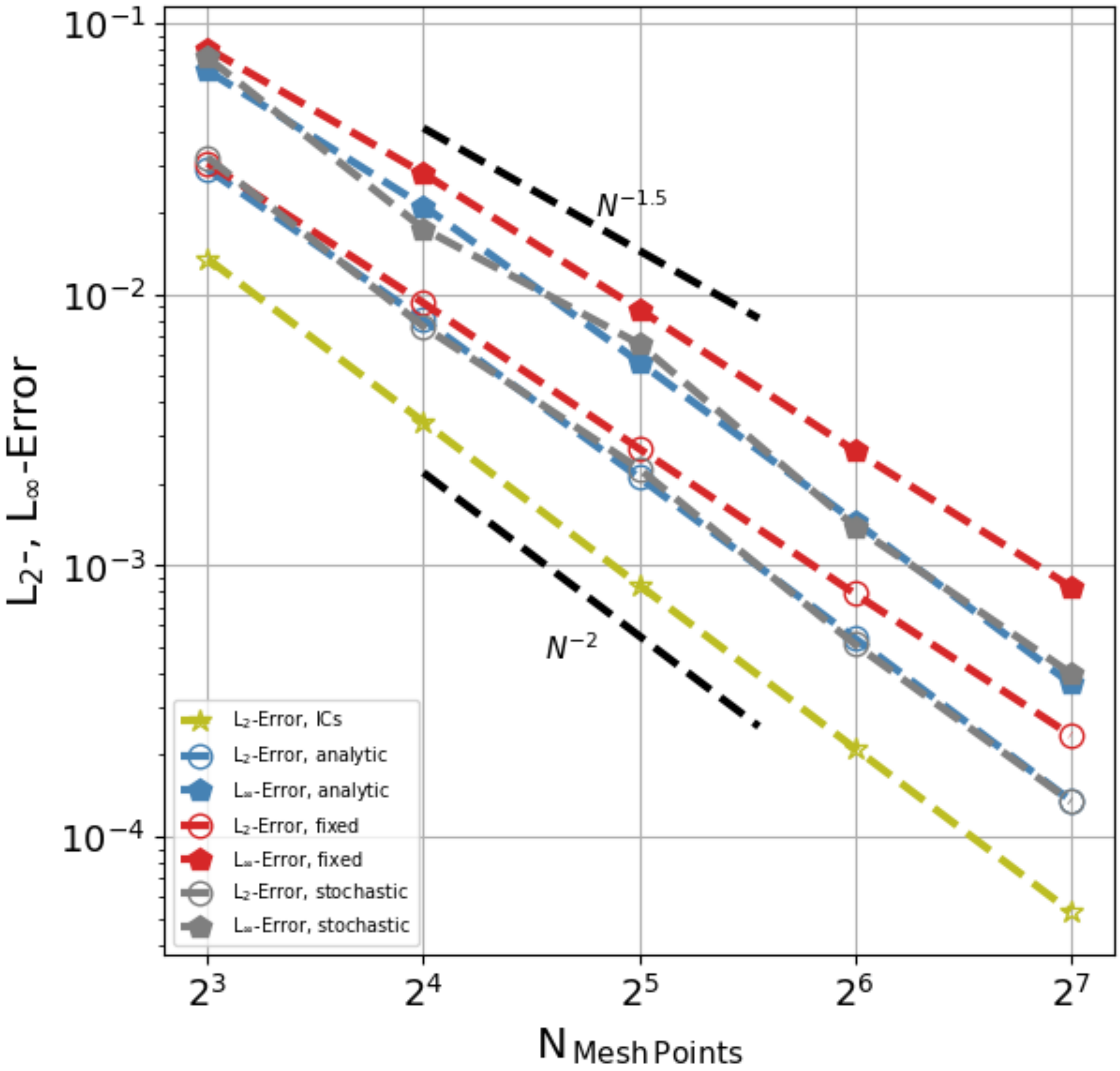

Figure S3. Convergence test II: L_2 and L_{inf} error norms for the 'analytic' (blue), 'fixed' (red) and 'stochastic' (gray) models. The plotted errors are averaged over a sample of 30 runs with different, randomly chosen values of the unperturbed thermodynamic parameters, (n, T, β). The L_2 error norm for the initial conditions (olive) is also shown together with curves representing N^{-2} and $N^{-1.5}$ error drop rate.
